# Advanced and explainable machine learning model for prediction of surface roughness of tempered steel AISI 1060

**DOI:** 10.1038/s41598-026-52458-y

**Published:** 2026-05-08

**Authors:** Firi Ziyad, Habtamu Alemayehu, Desalegn Wogaso, Getachew Semegn, Abdirahman Sheikhadan, Abreham Mulugeta, Adamu Hailu, Abdella Kereme

**Affiliations:** 1 College of Engineering and Technology, Kabridahar University, Kabridahar, Ethiopia; 2 Department of Mechanical Engineering, Haramaya Institute of Technology, Haramaya University, Haramaya, Ethiopia; 3 School of Mechanical & Industrial Engineering, College of Technology & Built Environment , Addis Ababa University, Addis Ababa, Ethiopia 1176,; 4 Department of Mechanical Engineering, Oda Bultum University, Chiro, Ethiopia; 5 Collage of Natural and Computational Science, Kabridahar University, Kabridahar, Ethiopia

**Keywords:** Machine learning, Surface roughness, Steel, Optimization, Engineering, Materials science, Mathematics and computing

## Abstract

This research examined the performance of a machine learning algorithm when predicting the surface roughness of tempered steel AISI 1060. Different machine learning algorithms, such as decision tree (DT), random forest (RF), adaptive boosting (ADB), gradient boosting (GB), and extreme gradient boosting (XGB), were optimized by using 10-fold cross-validation and the grid search method. From these optimized models, the decision tree, adaptive boosting, gradient boosting, and extreme gradient boosting were used as base models to develop a more powerful machine learning model called super learner machine learning. The linear regression (LR) was used as a meta-model in developing super learner machine learning. The developed super learner model performance was then validated against all machine learning models used in this research. For performance measurement metrics such as mean absolute error (MAE), root mean square error (RMSE), mean absolute percentage error (MAPE), and coefficient of determination (R²) has been used. The developed super learner model achieved the highest R² of 99.2% and the lowest MAPE of 2.6% on the test data set when compared with other machine learning models. Further, the SHAP method shows that hardness has the highest effect, followed by feed rate and cutting speed, respectively. Most machine learning approaches are not used practically for user applications, but in this research, a graphic user interface framework called fast, accurate, and intelligent (FAI) frame was developed to predict the surface roughness of tempered steel AISI 1060. This research is used for practical application for any user in industry and for research purposes.

## Introduction

Highly automated and intelligent machining systems are becoming more and more necessary in today’s environment. Machining is a manufacturing procedure that results in final dimensions, surface finish, and shape, among other things^1–3^. Surface roughness directly affects machined products’ ultimate shape and dimensional correctness. One crucial factor in determining the quality of a product is its surface roughness. Additionally, it shows the cost of machining, productivity, and efficiency^4,5^. The relationship between surface roughness and machining parameters such as tool wear, heat production, and ultimate product fatigue life is also present^6^. Because hardened steel is difficult to process with a standard cutting tool, it is machined using specialized tools like ceramic, coated, and cubic boron nitride. The construction, automotive, and defense industries all use hardened steel. In order to improve the product’s fatigue life and bearing capacity, hardened steel must have a low surface roughness^7^. Therefore, the product’s surface finish has a direct impact on the quality of hardened steel. A thorough understanding of the material qualities, cooling conditions, and machining parameters is necessary to achieve the lowest possible surface polish or roughness. Cutting speed, feed rate, depth of cut, tool size, cooling media, and workpiece hardness are some of the crucial machining factors. Understanding the relationship between surface roughness and various machining parameters is essential to achieving the desired surface finish. For example, modeling techniques can be used to determine the relationship between them^8^.

For modeling the surface roughness of hardened steel, numerous researchers have employed modeling methodologies such as the Taguchi method, response surface methodology, ANOVA, and regression model. For example^9^, modeled the surface roughness of hardened steel AISI 52,100 using artificial neural networks and response surface methods. Both models’ outputs exhibit a high degree of agreement with experimental data. The ANN outperforms the regression model in terms of prediction. The surface roughness of hardened steel EN31 can also be modeled using fuzzy logic; the model was developed using MATLAB 2020b. Fuzzy logic can forecast the surface roughness of EN31 steel based on the created model^10,11^. To forecast the surface roughness of hardened steel AISI 1060, the response surface approach, fuzzy logic, and simulated annealing (SA) were employed. Feed rates, hardness, and cutting speed were the machining parameters that were employed. RSM was used to get an R² of 99.64%. In a similar vein, RSM modeled the surface roughness of AISI 4340 hardened steel, and the Taguchi L27 technique was used to plan the experiment. Cutting speed, feed rate, and depth of cut are the input variables for this experiment. With an R2 score of 96.4%, the created model demonstrates that the RSM has a significant connection with experimental data^12,13^. When modeling hardened AISI 080A67 steel, the response surface approach was employed. When conducting an experiment, factors like cutting speed, feed rate, and depth of cut are taken into consideration. The Box-Behnken model is superior to the Box-Cox model, according to the results of two well-known response surface methodologies. The five coefficients of R2, R2 (Pred), R2 (Adj), percentage absolute error (PAE), and percentage square error (PSE) serve as the foundation for the comparison. Box-Behnken obtained an R² of 94.55%, an R² (Pred) of 12.79%, an R² (Adj) of 84.74%, a PAE of 8.79%, and a PSE of 1.42%, whereas the Box-Cox obtained an R² of 99.09%, an R² (Pred) of 85.42%, an R² (Adj) of 97.44%, a PAE of 2.26%, and a PSE of 0.18%.

It was evident from the literature research that more advanced modeling techniques are required because standard statistical models like Taguchi, RSM, complete factorial design, and regression models have incorrect conclusions^14^. Additionally, when modeling using this method, the majority of parameter combinations are not included. Therefore, a more sophisticated approach, like machine learning, is required. For example, when manufacturing hardened horses, researchers used machine learning to analyze their responses^15^. Several machine learning models, including gradient boost (GB), adaptive boost (AB), random forest (RF), and polynomial regression (PL), were used to estimate the hard turning of AISI D6 steel. In a similar vein^6^, predicted and optimized the surface roughness of hardened steel AISI 1060 under efficient cooling using supporting vector machines (SVM), response surface methodology (RSM), and genetic algorithms (GA). Dry and high-pressure coolant (HPC) cooling conditions were used in the experiment. According to the study’s findings, SVM is more accurate under HPC, while RSM is more accurate under dry machining. Furthermore, an artificial neural network (ANN) was used by^16^ to forecast the surface roughness of EN 24 T hardened steel. The ANN model produced a higher R² of 99.7% and a reduced root mean square error (RMSE)^17^. Used intelligent surface roughness prediction in inconel 625 milling using sensor fusion and explainable AI. SHAP analysis highlights depth of cut, feed, and entropy/spectral-center features as dominant contributors, aligning with known machining physics. The dataset showed maximum Ra = 0.62 μm, with T2 producing higher roughness than T1. The results indicate that the proposed interpretable pipeline maintains strong predictive performance while exposing process-relevant factors that can guide parameter selection and monitoring. Also^18^ used Assessment of Machining Performance for Intelligent Tool Wear Prediction Using Hybrid Extreme Learning Machine. Their study introduces a Hybrid Extreme Learning Machine (ELM) to predict tool wear during face milling of Inconel X750 using multiple sensors, aiming to optimize machining processes and extend tool lifespan^19^. Used SVM and ANN during classification of surface roughness for CNC face milling of Inconel 625 superalloy utilizing cutting force signal features.

The current machine learning model needs a lot of data to train effectively, generalize, and be applied to unseen data, despite the fact that numerous artificial intelligence and machine learning techniques are employed to forecast the surface roughness of hardened steel. Large data collection is expensive and time-consuming. In addition to this, obtaining a lot of data through experimentation requires a lot of cooling media, which has negative effects on the environment and human health. The use of more potent machine learning techniques, such as ensemble learning, physics-informed machine learning, and traditional neural networks for data production, is required to close this gap.

To solve a single machine learning problem, ensemble machine learning uses various machine learning as base learners. It made use of single-base machine learning’s prediction ability. In engineering, ensemble machine learning is used to address non-linear complex problems. For example^20^, predicted tool condition monitoring using ensemble machine learning. The surface roughness and tensile strength of additively manufactured polylactic acid (PLA) were modeled using a variety of ensemble machine learning techniques, including adaptive boosting (ADB), random forest (RF), gradient boosting (GB), extreme gradient boosting (XGB), and extremely randomized tree (XRT)^21^. It was also applied to the detection of faults^22^. The stacking ensemble, K-nearest neighbor, and Naïve Bayes algorithm were employed. Adaptive boosting ensemble tree was employed to enhance the current ensemble model^23^ and is applied in the industry’s maintenance process. In a similar vein^24^, suggested including an explainable AI (XAI) framework for machine learning and ensemble deep learning^25^. Used extreme gradient boosting for prediction of surface roughness of mild steel AISI 1060.

Although ensemble machine learning is widely utilized in the manufacturing sector, its employment in hardened steel AISI 1060 machining is not predetermined. Additionally, because machine learning models cannot be interpreted, they are referred to as “black boxes.” This study uses stacking ensemble machine learning, also known as super learner machine learning, to forecast the surface roughness of hardened steel AISI 1060 in order to close this gap. Super learner machine learning was previously utilized by researchers like^26^ to estimate the surface roughness of tempered steel AISI 1060 under efficient cooling. However, their model is not appropriate when it comes to real applications. To overcome their limitations, our research created a useful application known as the FAI framework. This framework is utilized for any user’s practical application. To improve its interpretability, this study also uses a SHAP approach method.

The novelty of this research lies in the development of a high-fidelity predictive framework specifically for the dry turning of tempered AISI 1060 steel. While prior studies have explored super-learner applications in cooled environments, the physical interactions at the tool-chip interface in dry machining are fundamentally distinct due to the lack of heat dissipation and lubrication. This study provides the first SHAP-based diagnostic of how material hardness dictates surface topography when subjected to the intense thermal loads characteristic of dry operations. By isolating the impact of bulk hardness in a non-lubricated environment, this work offers unique scientific insights into the self-lubricating or abrasive behaviors of tempered phases that are otherwise masked in cooled machining studies. We emphasize that the novelty of this work extends beyond the model comparison to the original development of a task-specific GUI framework for dry machining. While high-performance models like XGB and Super Learners exist in literature, they are often inaccessible to shop-floor practitioners due to their computational complexity. This study provides a novel, standalone intelligent system that encapsulates the Super Learner. By integrating the SHAP-derived insights into a functional interface, we provide a tool that can predict Ra in dry turning without requiring the user to have expertise in machine learning, which represents a distinct advancement in Industrial 4.0 readiness for sustainable manufacturing.

The novelty of this research, distinguishing it from traditional RSM, ANN, and standalone XGBoost studies on similar steels, is three-fold. First, it investigates the sensitivity of surface roughness to a wide range of tempered hardness levels (40-56 – HRC) specifically under dry machining conditions, where the absence of lubrication intensifies the impact of material properties on surface finish. Second, while prior studies treat the model as a black box, this work employs SHAP (SHapley Additive exPlanations) to provide interpretation of how feed rate and hardness interact to dictate surface quality. Third, the study introduces a novel, task-specific GUI framework that deploys the Super Learner model as a standalone tool, effectively bridging the gap between high-level ensemble modeling and practical industrial application.

## Materials and methodology

In this section the material used for machining purposes, the cutting tool used, the machining environment, and the machine learning used are discussed.

### Machine and equipment’s

**Fig. 1:**
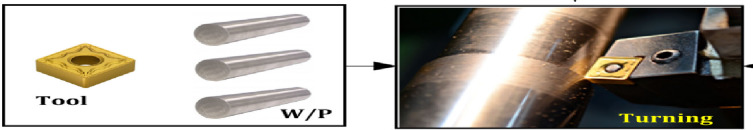
The experimental work^16^.

This study used a powered centered lathe (model number CS6266, China, power = 7.5 KW, maximum swing over bed = 660 mm, maximum workpiece length = 2500 mm) to conduct the experiment. An ISO-designated coated carbide insert (SNMG 120408) was the cutting instrument. TiCN was the coating substance utilized. Co. and WC made up the tool. The tool’s rake and clearance angles are both 00. ISO PSBNR 2525 M12 is the specification for the tool holder. Table 1 lists experimental conditions. Figure 1 displays the experimental configuration.

**Table 1:** Experimental condition^16^.

Categories	Specification
Workpiece	AISI 1060
Machine tool	Lathe (china), power, 7.5KW
Dimension of workpiece	Length= 200 mm, external diameter= 120 mm, internal diameter= 45 mm
Treatment	Thermally: austenitizing, tempering and quenching
Hardness	Before = 89 Rockwell B, after = 40, 48, 56 Rockwell C
Workpiece composition	Fe = 98.6%, Mn = 0.7%, C = 0.6%, S = 0.05%. *P* = 0.04%, others = 0.01%
Workpiece physical properties	Density = 7.85 g/cm3, melting point=1510 °C, ultimate strength= 620 MPa, yield strength= 485 MPa, modulus of elasticity=210GPa, passion ratio = 0.30
Cutting tool	SNMG 120,408 (ISO specification)
Geometry of cutting tool	Clearance angle = 0^0^, rake angle = 0^0^, shape=square, noise radius = 0.8 mm, effective cutting edge length = 11.91 mm, insert thickness = 4.76 mm, chip breaker double side
Coating and core	TiCN, and WC, Co
Tool holder	PSBNR 2525 M12 (ISO specification)
Cutting speed(Vc)	Min = 58 m/min, max = 161 m/min
Feed rate(F)	Min = 0.1 mm/rev, max = 0.16 mm/rev
Depth of cut	1.0 mm

### Data used for research

The database used for this research was taken from^16^, specifically isolating the dry turning subset of tempered AISI 1060 steel and its statistical detail was shown in Table 2. The dataset consists of 48 unique experimental runs (3 speeds × 3 feeds × 3 hardness levels).

The cutting speed (Vc) used was in the range of 58 m/min-161 m/min, the feed rate (F) used was in the range of 0.1 mm/rev-0.16 mm/rev, and the hardness (H) used was in the range of 40HRC-56HRC.40 .

The secondary analysis of this data is justified because the original study primarily focused on cooling performance using RSM and SVR. Our research introduces a Stacked Super Learner framework that captures complex non-linearities missed by traditional RSM. Furthermore, by applying SHAP interpretability and developing a functional GUI, this work transitions the data from a comparative study of coolants to a dedicated decision-support system for dry machining, providing new scientific value regarding the material’s behavior under high-friction, non-lubricated conditions. The selection of this dataset allows for a direct comparison between traditional statistical modeling (RSM) and advanced ensemble learning (Super Learner). While the original study used this data to evaluate cooling efficiency, the current work focuses on the predictive modeling of dry machining, where thermal loads are highest. Using 48 data points in conjunction with a 10-fold cross-validation strategy ensures that the Super Learner framework remains stable and avoids overfitting, providing a reliable basis for the development of the proposed GUI-based decision support system.

**Table 2:** Data used for this research.

Parameters	STD	Mean	Min	Max	Q1	Q2	Q3
Ra	0.436	1.326	0.73	2.35	0.98	1.15	1.85
Vc	34.25	103.73	58	161	67.75	98	149.5
F	0.02	0.13	0.1	0.16	0.15	0.13	0.155
H	5.333	48	40	56	40	48	56

Max=maximum, Min=Minimum, STD= Standard deviation, Q1, Q2, Q3 = 25th, 50th, 75th percentiles.

### Machine learning models

- Decision tree (DT).

Decision trees are supervised machine learning algorithms with a tree-like flow and structure that address both regression and classification problems. Using the decision rules it learned from the training dataset, the decision tree model predicts the value of the target variable. The node’s root is where predictions begin, and the characteristics of the roots are compared. Based on the comparison, the branch passes to the next node and advances to the relevant value of the node characteristics. The basic steps in a decision tree are displayed below^27^.

- Start with the root node and original dataset as S.
- It searches the next attributes from unselected attributes of set S during every iteration and calculates entropy [EP] and information gain G[I] by using this formula shown below.

1 $$\:\mathrm{E}\left(\mathrm{P}\right)\:=-{\sum\:}_{i=1}^{n}{p}_{i}*{\mathrm{log}}_{2}{p}_{i}$$

2 $$\:\mathrm{G}\left(\mathrm{I}\right)\hspace{0.17em}=\hspace{0.17em}\mathrm{E}\left(\mathrm{P}\right]-{\sum\:}_{I=1}^{n}\frac{{n}_{i}}{n}E\left(i\right)$$

- The largest information gain or smallest entropy attribute is selected.
- A subset of data is created by the set of S, split by the selected attribute.

The algorithm continues to recur on each subset and consider only the attributes that were never selected before.

b) Random forest (RF).

**Fig. 2:**
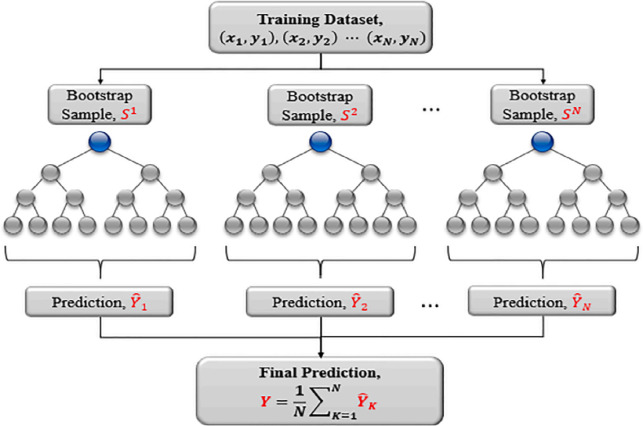
Random forest model^30^.

Multiple base learners were utilized in the random forest model to solve problems and improve model performance^28^. To create a decision tree forest, this ensemble-based machine learner uses bootstrap samples from the training dataset^29^. The decision node and the leaves in this model explain the decision tree, while the data is split at the decision node, which represents the final result. Because of its versatility and ease of use, the random forest model is frequently employed in regression and classification applications. Figure 2 illustrates the random forest model’s constriction split.

c) Adaptive boosting (ADB).

A single decision tree is known as a weak learner because of its limited scope. In ADB model, multiple weak learners can be combined to create a strong learner^31,32^. Devised the architecture for the boosting method in 1990, which successively combines several weak learners, and confirmed this idea. Equation^3^ shows that just the strongest tree is added to the total each time a new tree model is introduced, keeping the general tree out. In this way, when iterative calculations are made, the overall model performance will gradually improve. However, something is not right here. After obtaining the initial basic tree model, some samples in the data set are correctly identified, while others are classed wrongly. The ADB algorithm, a simple weak classification algorithm enhancement technique, improves data classification capability by continuous training. Once the initial weak classifier is learned from the training samples, the incorrect samples are combined with the untrained data to form a new training sample. The second weak classifier is also obtained by learning this dataset. By mixing the wrong sample with the untrained data, a new training sample is formed, which may be used to train the third weak classifier. Before we can finally generate the improved, robust classifier, this process needs to be carried out several times.

**Fig. 3:**
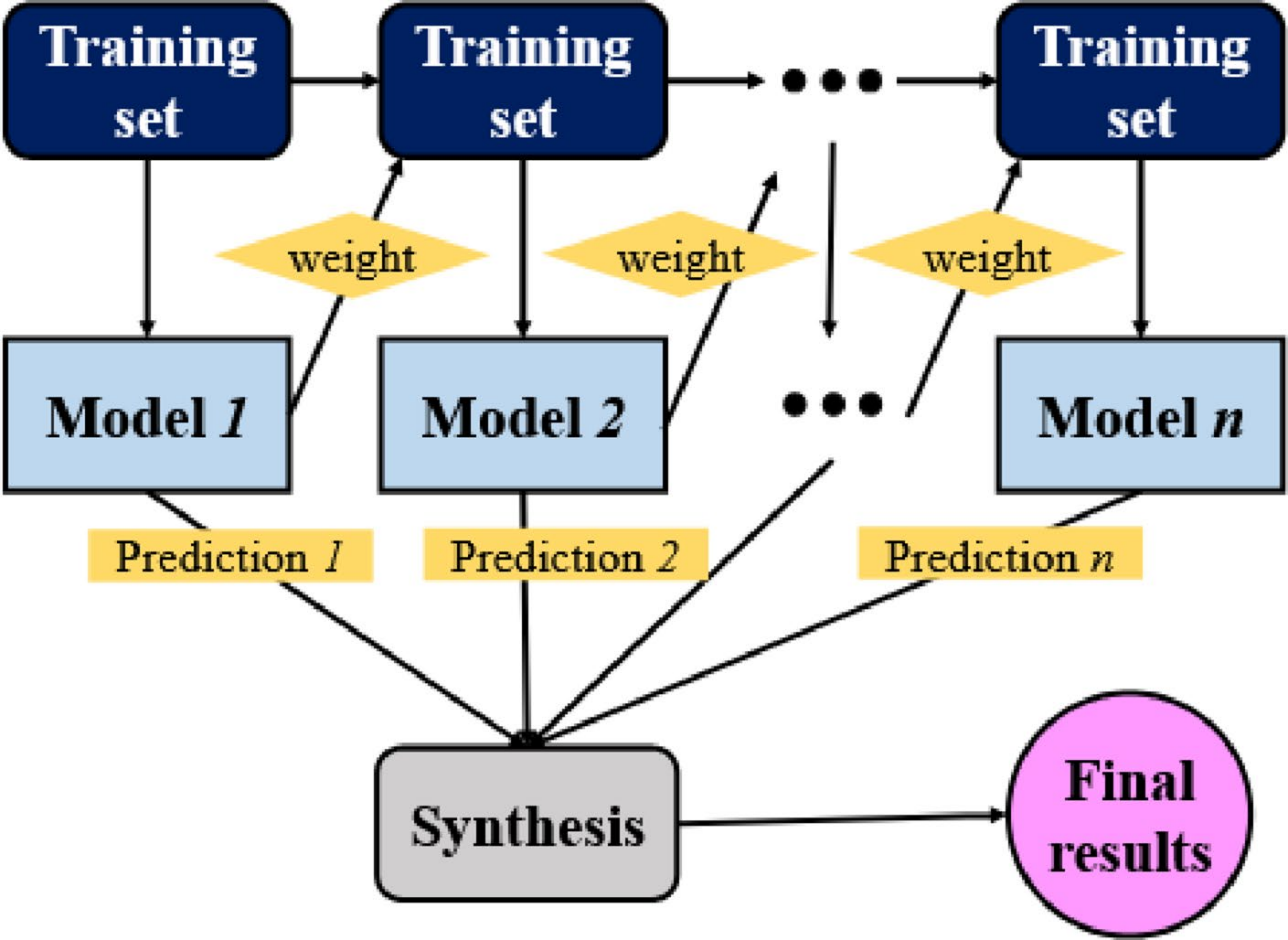
Adaptive boosting weight calculation process^34^.

To increase the number of accurate classifications, the ADB approach gives the samples different weights^33^. Since the mistakenly classified samples must be raised while the correctly identified samples are given relatively low weights, the model is compelled to concentrate more on the incorrectly categorized samples^31^. Figure 3 illustrates the general calculating process of the ADB algorithm. When training each basic tree model, the weight distribution of each sample in the data set must be changed. Because every training data set is different, training results will differ, and in the end, all of the results are combined^34^.

.

3 $$\:{F}_{n}\left(x\right)={F}_{m-1}\left(x\right)+{argmin}_{h}{\sum\:}_{i=1}^{n}L\left({y}_{i},{F}_{m-1}\left({x}_{i}\right)+h({x}_{i}\:\right))$$

Where yi is the i-th tree’s prediction result, h(xi) is the recently inserted tree, Fn(x) is the overall model, and Fn-1(x) is the overall achieved in the previous round.

d) Gradient boosting (GB).

Gradient boosting create more powerful learner by combining weak base learner just like adaptive boosting. By using gradient decent method it correct the limitation of weak models. The model initialized by constant value^35^. Eg (4).

4 $$\:{F}_{0}\left(x\right)=\frac{\mathrm{a}\mathrm{r}\mathrm{g}\:\mathrm{m}\mathrm{i}\mathrm{n}}{{\upgamma\:}}{\sum\:}_{I=1}^{N}L\left({Y}_{i},\right)$$

The base learner $\:{h}_{t}\left(x\right)\:$was fitted by the GB to previous pseudo residual$\:{\gamma\:}_{i,\:t}$ at the t^th^ step as shown in E q (5) and transform model to Eq. (6)

5 $$\:{\gamma\:}_{i,\:t}\:=-{\left[\frac{\partial\:L({Y}_{i},\:\:F\left(\:{X}_{i}\right))}{\partial\:F\left(\:{X}_{i}\right)}\right]}_{F\left(X\right)={F}_{t-1\left(X\right)}},\:\mathrm{f}\mathrm{o}\mathrm{r}\:\mathrm{i}\hspace{0.17em}=\hspace{0.17em}1,\dots\:..,\:\mathrm{N}\:$$

6 $$\:{F}_{t}\left(X\right)={F}_{t-1\left(X\right)}+{\gamma\:}_{t}{h}_{t}\left(X\right),\:for\:t=1,\dots\:\dots\:..,\:T$$

T is base learner number, $\:{\gamma\:}_{\:t}$ is the multiplier and determined from the found single solution to single variable optimization as shown in Eq. (7).

7 $$\:{\gamma\:}_{t}\:=\:{arg}_{\gamma\:}min{\sum\:}_{i=1}^{N}L({Y}_{t},\:{F}_{t-1\left({X}_{i}\right)}\:+\:{\upgamma\:}{h}_{t}\left({X}_{i}\right))$$

Where $\:\gamma\:$ ϵ [0, 1] is the learning rate^35^.

e) Extreme gradient boosting.

To apply the gradient boosting more efficiently, the branch of the boosting model called extreme gradient boosting (XGB) was developed^36^. To modify the gradient boosting, extreme gradient boosting adds a regularization component to its objective function to overcome the fitting problem. As shown in Eq^8^., the XGB increased generalizability and brought a highly accurate model by applying the regularization term^37^.

8 $$\:\mathrm{O}\mathrm{b}\mathrm{j}\left(\Theta\right)\:=\:{\sum\:}_{i=1}^{N}L({Y}_{i},\:{\rm \hat{Y}}_{i}+{\sum\:}_{t=1}^{T}\varOmega\:({f}_{t\:}))$$

Where L (⋅) is the training loss between the predicted ($\:{\rm \hat{Y}}$) and actual (Y) values of the response and where $\:\varOmega\:$ (⋅) represents the regularization term outlined in Eq. (9):

9 $$\:\varOmega(f_{t})=\upgamma\:{\rm M}+\frac{1}{2}\lambda\parallel\omega_{t}\parallel^{2}$$

where γ and λ are the penalty coefficients, M represents the number of leave nodes in the tree, and $\:{\omega\:}_{t}\:$ denotes the leaf weights.

### Super learner model

To build a single, more powerful model, the super learner approach combines multiple machine learning models or base learners. Using K-fold cross-validation (with K = 10 in this case), the best combination of base learners is determined by assigning each base learner a distinct weight, as decided by the algorithm. The super learner model method was applied in this study using the following steps:

1. The training data is first divided into 10 folds.
2. In the second step, a group of strong models is selected to serve as candidate base learners. These models are.
3. Individually optimized before being used in the super learner architecture.
4. The model is refined by fitting it to the complete training dataset in the third step, where the performance of each candidate base learner is evaluated using tenfold cross-validation, and a matrix of predictions is generated during the validation process.

In this study, the potential base models for the super learner method were decision tree (DT) gradient boosting (GB), and extreme gradient boosting (XGB), with linear regression (LR) acting as the meta-model.

The selection of base learners for the Super Learner framework was guided by the need for high predictive accuracy and robustness against overfitting in a small-sample experimental environment (*n* = 48). Decision Trees (DT**)** and Boosting Ensembles (ADB, GB, XGB) were chosen because tree-based architectures are inherently suited for tabular data, as they partition the feature space into hierarchical segments that align with the physical thresholds of machining parameters (Speed, Feed, Hardness). While Support Vector Machines (SVM) are effective for small datasets, Our goal was to advance the state-of-the-art by employing boosting-based ensembles, which are more capable of capturing the complex, non-linear gradients of surface topography in dry machining.

Although several ensemble models were optimized, the final selection for base learners focused on architectural diversity. Random Forest (RF) was excluded from the final stacking protocol because its contribution to the meta-model was found to be redundant alongside the Gradient Boosting (GB) and XGBoost models. By selecting a leaner pool of diverse models (DT, ADB, GB, and XGB), the Super Learner avoids the risk of overfitting through redundant feature mapping. This ensures a more stable meta-model (Linear Regression) that generalizes better to the extreme thermal variations of dry machining. The stacking protocol utilized a 10-fold Out-of-Fold (OOF) prediction strategy. The training set was divided into 10 subsets; four base models (DT, ADB, GB, and XGB) were trained on nine subsets to predict the tenth. This process was repeated until OOF predictions were generated for the entire dataset. These predictions served as the input features for the Linear Regression meta-model. This specific sequence prevents ‘data leakage,’ as the meta-model is trained on predictions for data points that the base models did not see during their specific training fold.

Linear Regression was selected as the meta-model to maintain the interpretability of the stacking framework. Using a complex non-linear meta-model on a small dataset often leads to ‘meta-overfitting.’ Linear Regression effectively acts as a weighted averaging mechanism, assigning coefficients to the most reliable base learners while keeping the final prediction stable and physically grounded.

### Cross validation and hyperparameter tuning

The values of a model’s hyperparameters determine its predictive ability and generalization potential. The optimal hyperparameter settings are found by hyperparameter tuning or optimization. Grid search is a well-liked technique for optimizing hyperparameters. To prevent overfitting problems, a K-fold cross-validation technique is applied during the hyperparameter tuning process. Separating the dataset into training and testing datasets, which account for 80% and 20% of the final dataset, respectively, is the first stage. K-fold cross-validation is then performed.

The rationale for avoiding a separate static validation set is the limited size of the experimental dataset (*n* = 48). In such ‘small-data’ scenarios, a three-way split (Train/Validation/Test) would starve the learning process of critical physical information. Instead, 10-fold cross-validation was integrated within the training phase to serve as an internal validation mechanism for hyperparameter tuning and meta-model stacking. This ensured that the model remained generalized without sacrificing training data.

**Fig. 4:**
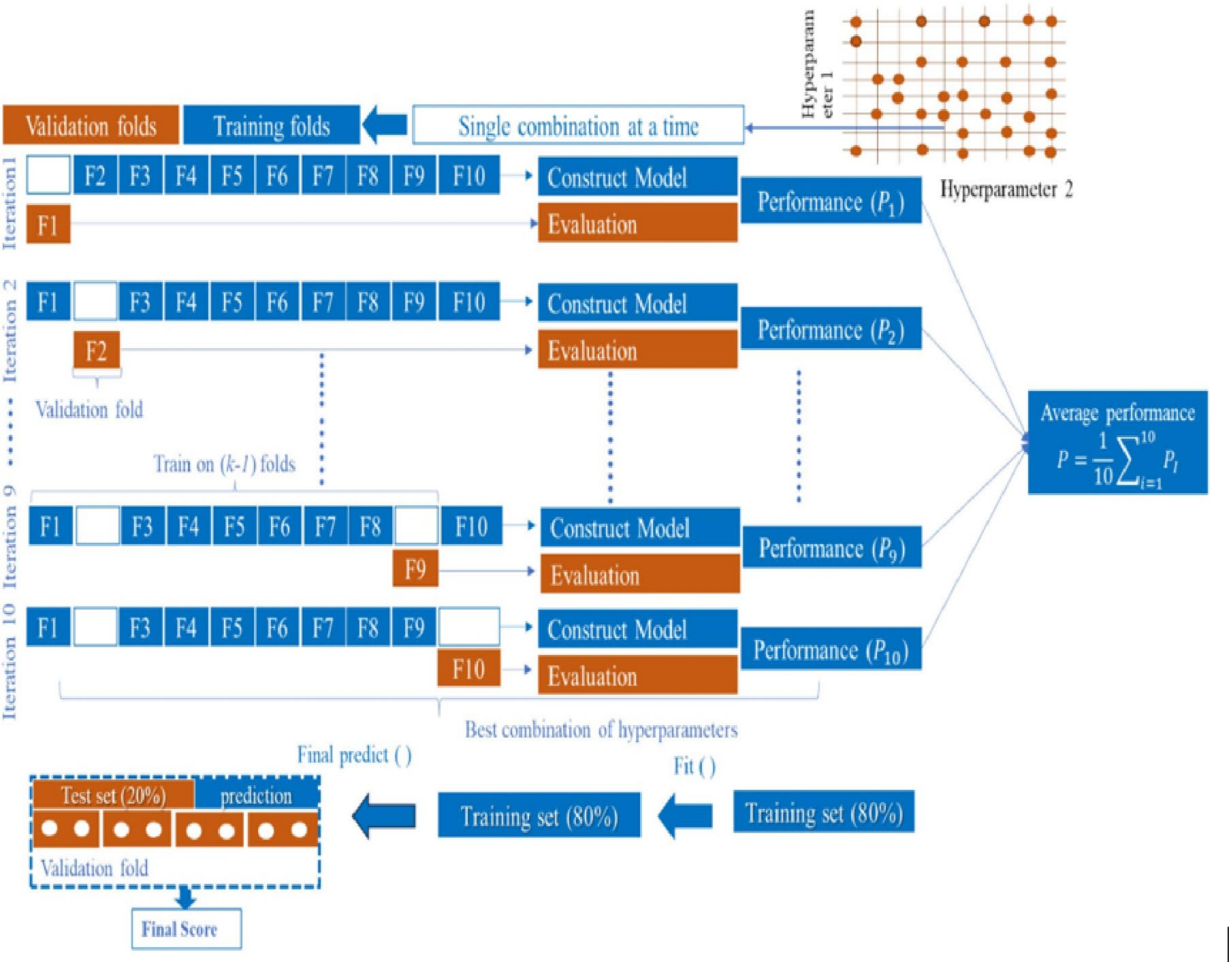
cross valiadation and hyperparameter tuning.

**Fig. 5:**
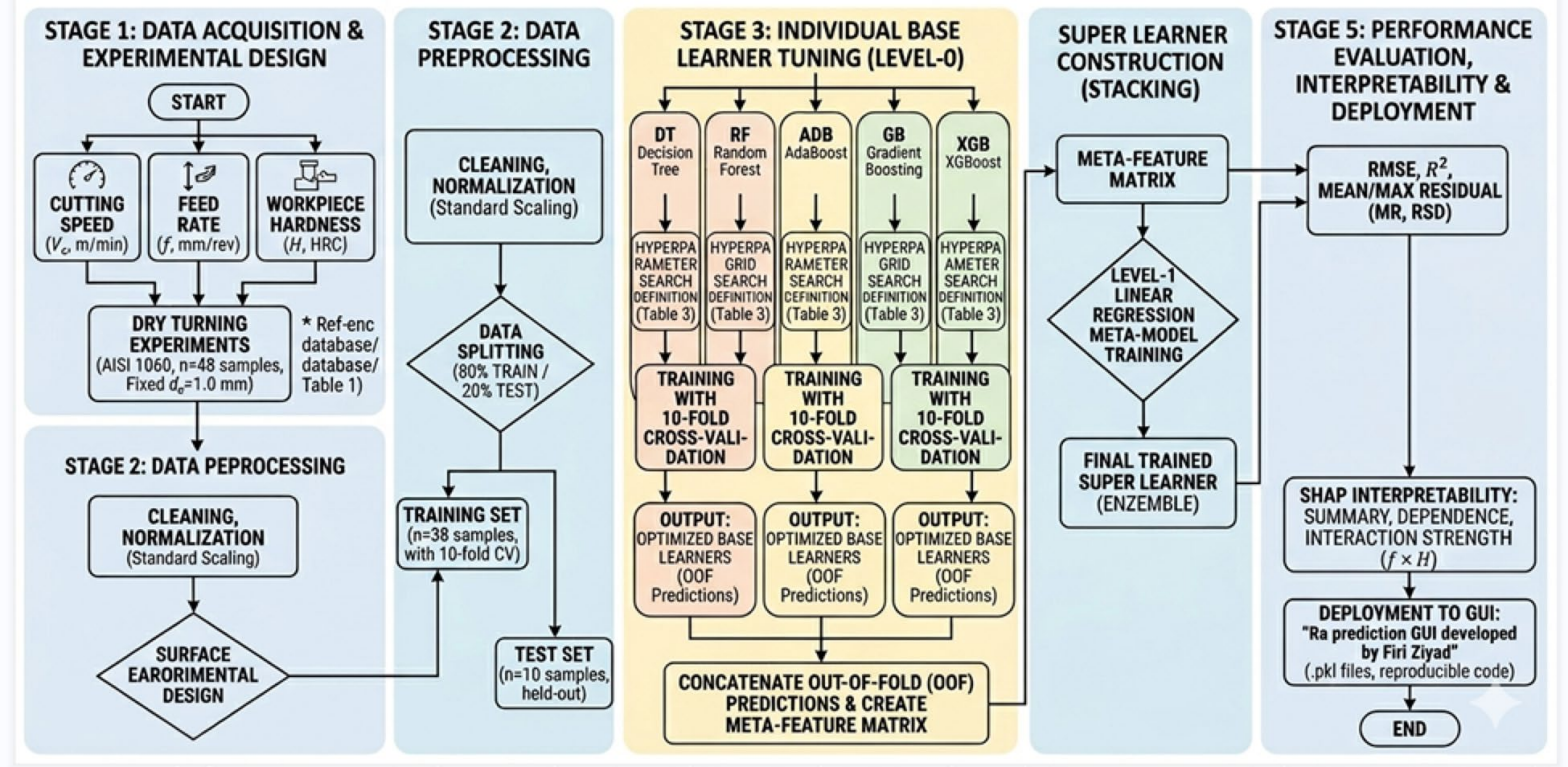
Flow chart.

A common challenge in experimental machining studies is the trade-off between model complexity and dataset size. For this study (n = 48), a traditional three-way split involving a separate validation set was deemed suboptimal as it would severely limit the training observations available to capture the non-linearities of dry-turning physics. Consequently, a 10-fold Cross-Validation (CV) approach was integrated within the training phase. This methodology effectively utilizes the CV folds as an ‘internal validation mechanism’ for hyperparameter tuning and meta-model stacking, while the 20% hold-out set remains strictly reserved for an unbiased final performance evaluation. This hybrid approach ensures high performance that avoids both data scarcity and information leakage. Figure 4 shows K-fold cross validation used in this study and Fig. 5 shows all methodology used in this study.

### ML performance measures

To evaluate the accuracy of the ML models, four distinct statistical measures are employed: mean absolute percentage error, root mean squared error, coefficient of determination, and mean absolute error. The mathematical expression for these metrics is as follows (refer to Eqs. (10)–(13)).

10 $$\:MAPE=\frac{1}{N}{\sum\:}_{i=1}^{N}\left|\frac{{Y}_{i-}{\rm \hat{Y}}_{i}}{{Y}_{i}}\right|$$

- Root mean squared error (RMSE):

11 $$\:RMSE=\sqrt{\frac{1}{N}{\sum\:}_{i=1}^{N}{({Y}_{i}-{\rm \hat{Y}}_{i})}^{2}}$$

- Mean absolute error (MAE):

12 $$\:MAE=\frac{1}{N}{\sum\:}_{i=1}^{N}\left|{Y}_{i-}{\rm \hat{Y}}_{i}\right|$$

- Coefficient of determination, R2:

13 $$\:{R}^{2}=1-\frac{{\sum\:}_{i=1}^{N}({Y}_{i}-{\hat{Y}}_{i})}{{\sum\:}_{i=1}^{N}({Y}_{i}-{\stackrel{-}{Y}}_{i})}$$

## Results and discussion

### Hyperparameter optimization results

Several performance metrics, including MAE, RMSE, MAPE, and R2, have been used to evaluate the models’ ability to predict surface roughness. The effectiveness of machine learning techniques like DT, RF, ADB, GB, and XGB has been assessed by looking at the results of various measures. Grid search and 10-fold cross validation are utilized to adjust the hyperparameters of these models, with RMSE serving as the assessment metric. Table 3 displays the improved hyperparameter results. For DT, the maximum tree depth was 5. The optimum settings for RF were determined to be 1 minimum sample leaf, 2 minimum sample splits, 30 maximum depth of tree, and 100 estimators. For ADB, the optimum parameters was a learning rate of 1 and a number of estimators of 100. In the case of GB, the learning rate is 0.1, the maximum depth of the tree is 1, and the number of estimators is 100. For XGB, the optimum parameters are a learning rate of 0.1, 100 number of estimators, and a maximum tree depth of 10.

Finally, based on the optimum parameters, the optimized DT, ADB, GB, and XGB integrated by using linear regression (LR) as a meta-model were.

**Table 3:** Optimized hyperparameters.

Models	Hyperparameters	Search space	Optimized value
DT	Maximum depth of tree	[1 20]	5
RF	Number of estimator Maximum depth of tree Minimum sample leaf Minimum samples split	[10 500] [1 100] [1 30] [1 20]	100 30 1 2
ADB	Number of estimator Learning rate	[10 500] [0.001 1]	100 1
GB	Number of estimator Maximum depth of the tree Learning rate	[50 1000] [1 10] [0.01 0.2]	100 1 0.1
XGB	Number of estimator Maximum depth of the tree Learning rate	[10 1000] [1 10] [0.01 0.2]	100 10 0.1

### Model performance

**Fig. 6:**
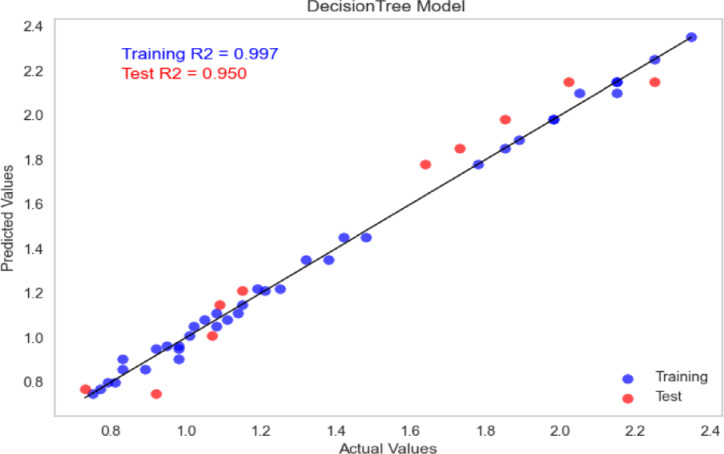
Scatter plot of DT.

**Fig. 7:**
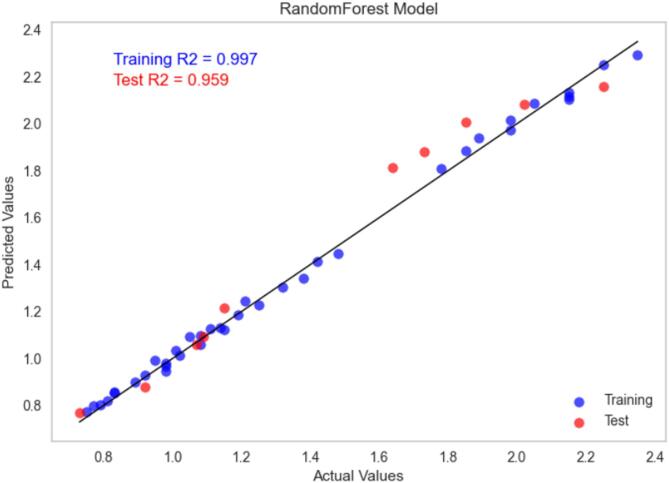
Scatter plot of the RF.

**Fig. 8:**
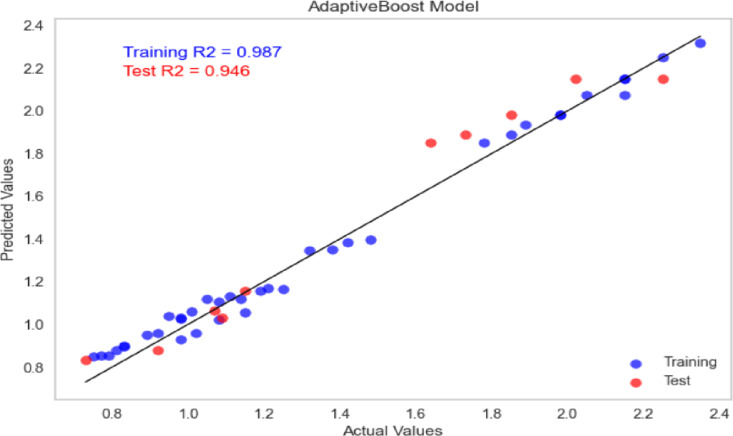
Scatter plot of ADB.

**Fig. 9:**
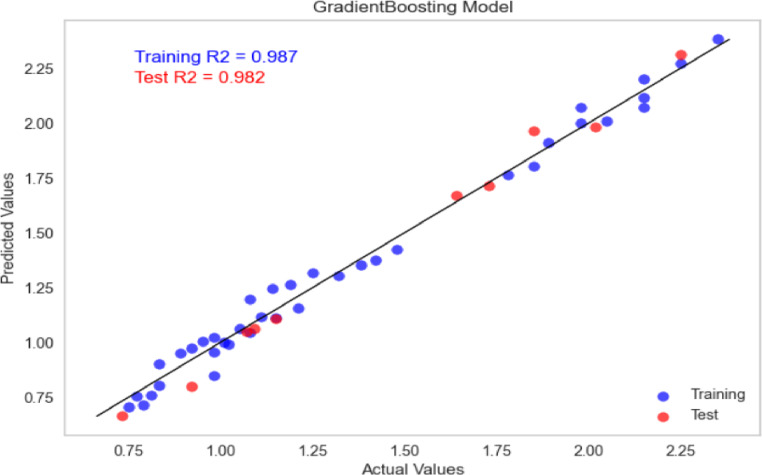
Scatter plot of GB.

**Fig. 10:**
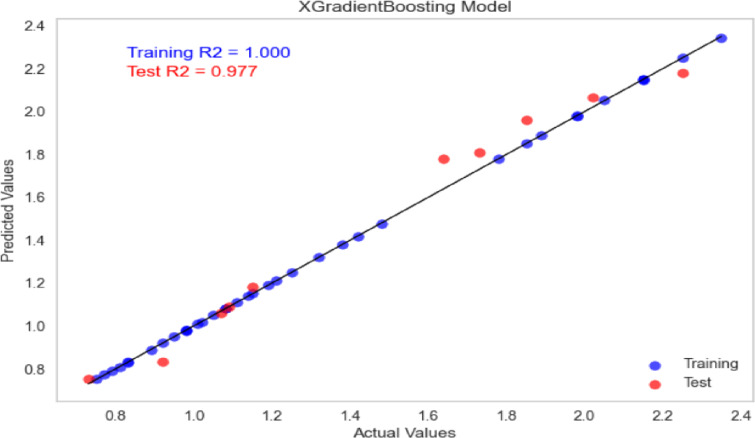
Scatter plot of XGB.

**Fig. 11:**
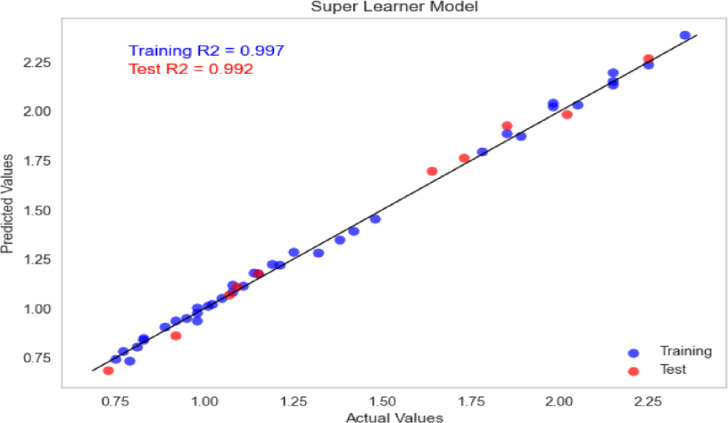
Scatter plot of the super learner model.

The performance of machine learning used in this research has been shown on a scatter plot. The scatter plot shows the relationship between experimental and predicted surface roughness. Based on the scatter plot of Figs. 6, 7, 8, 9, 10 and 11, the model shows R² > 98% and R² > 94% for both the training and testing datasets. This shows our models DT, RF, ADB, GB, XGB, and super learner models have high prediction capabilities. The advantage of using ensemble machine learning models and the super learner model is to reduce error in predicting surface roughness and to increase prediction capability. As shown from the scatter plots of Figs. 6, 7, 8, 9, 10 and 11, all models have predicted surface roughness, which is closely related to 45^0^. If all points are closely scattered and related to 45^0^ on the scatter plot, it shows accuracy, and if not, it shows no accuracy.

**Fig. 12:**
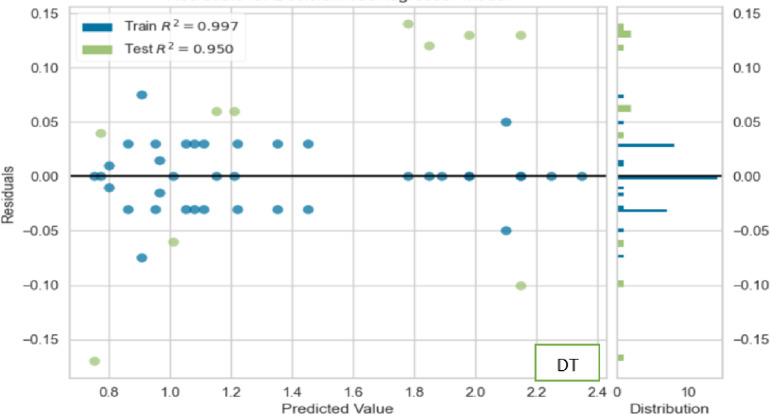
Resedual plot of the DT.

**Fig. 13:**
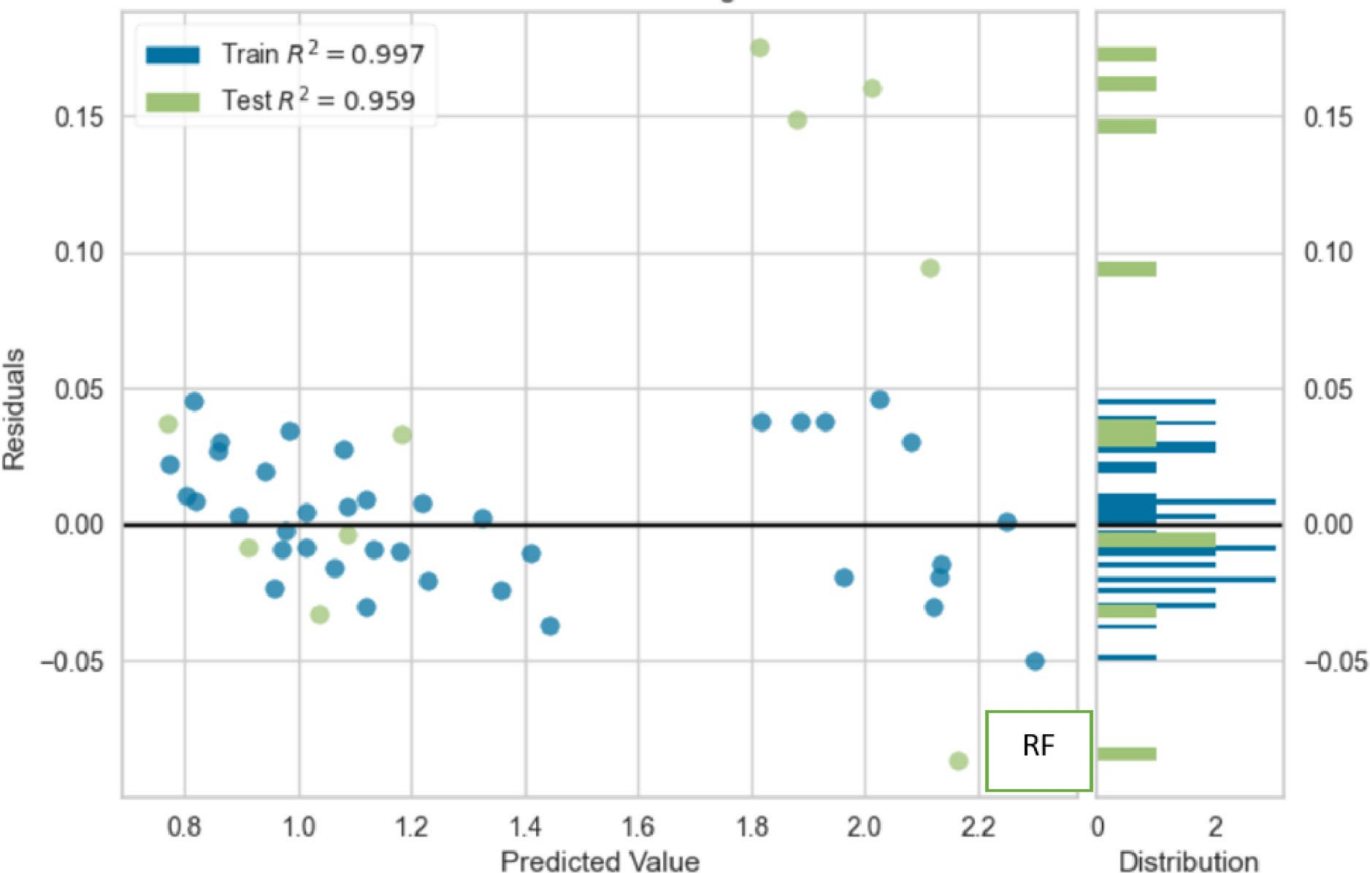
Resedual plot of RF.

**Fig. 14:**
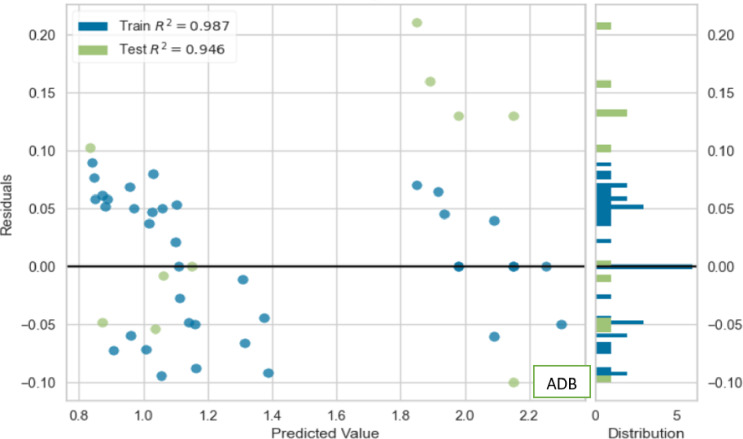
Resedual plot of the ADB.

**Fig. 15:**
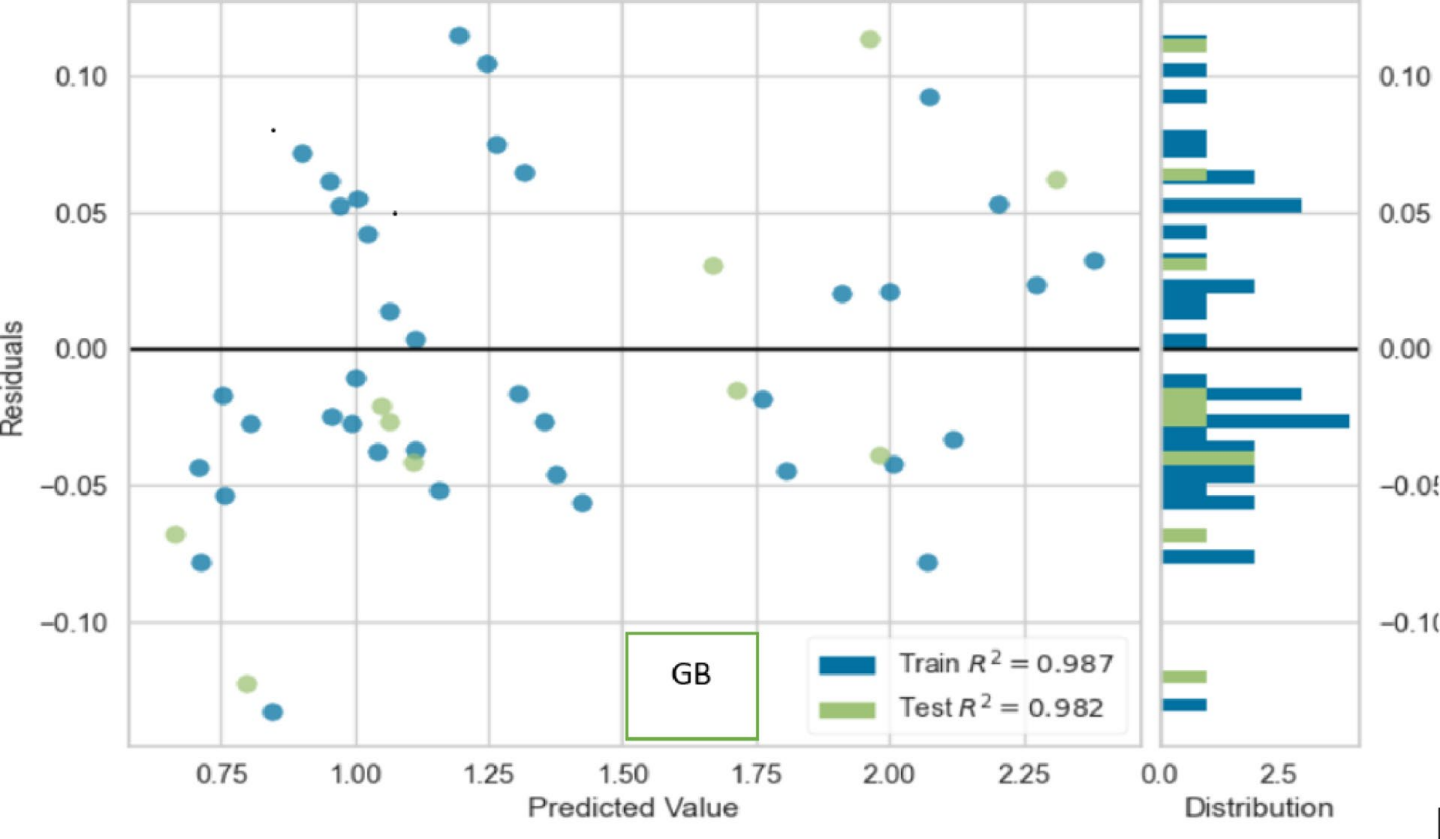
Resedual plot of GB.

**Fig. 16:**
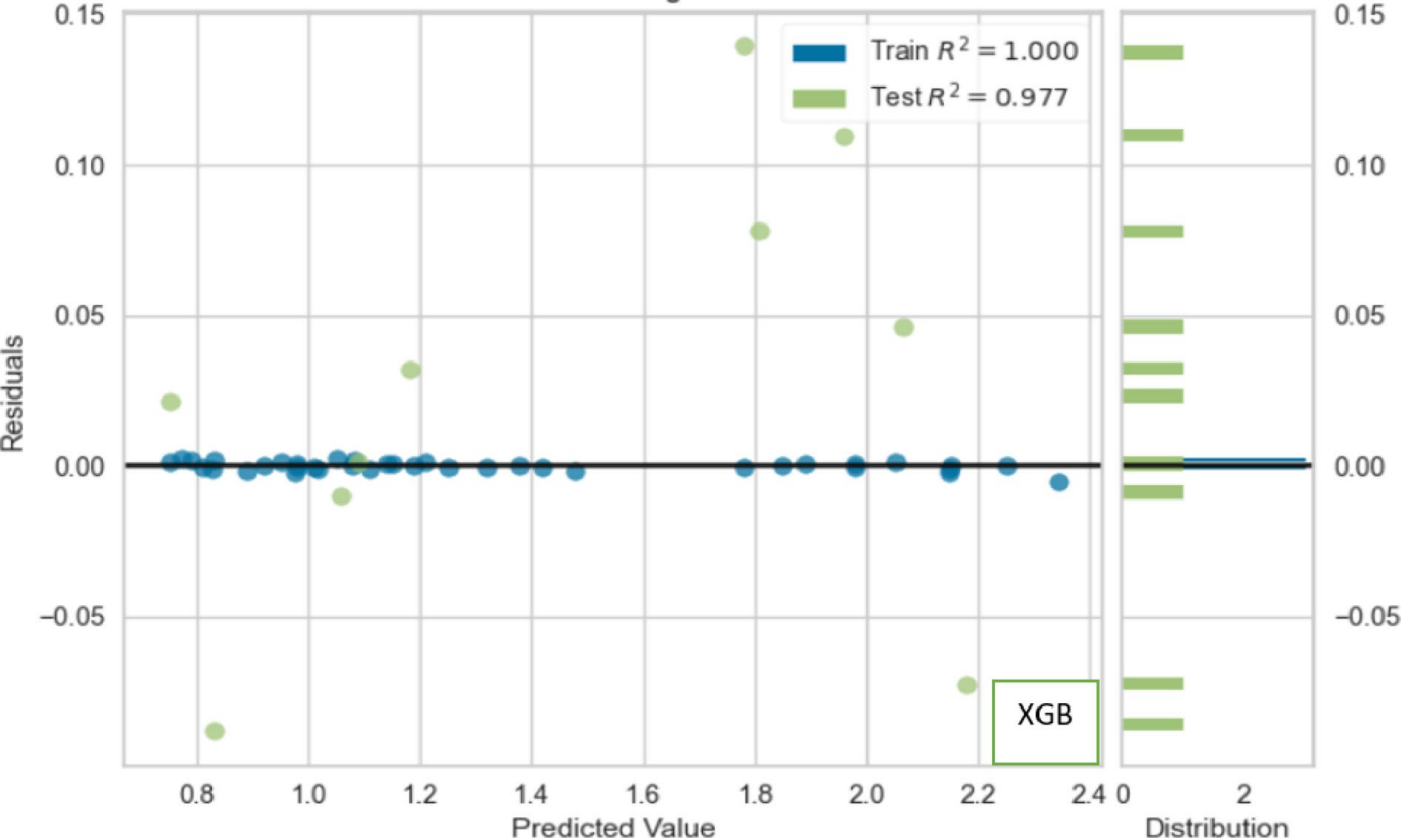
Resedual plot of XGB.

**Fig. 17:**
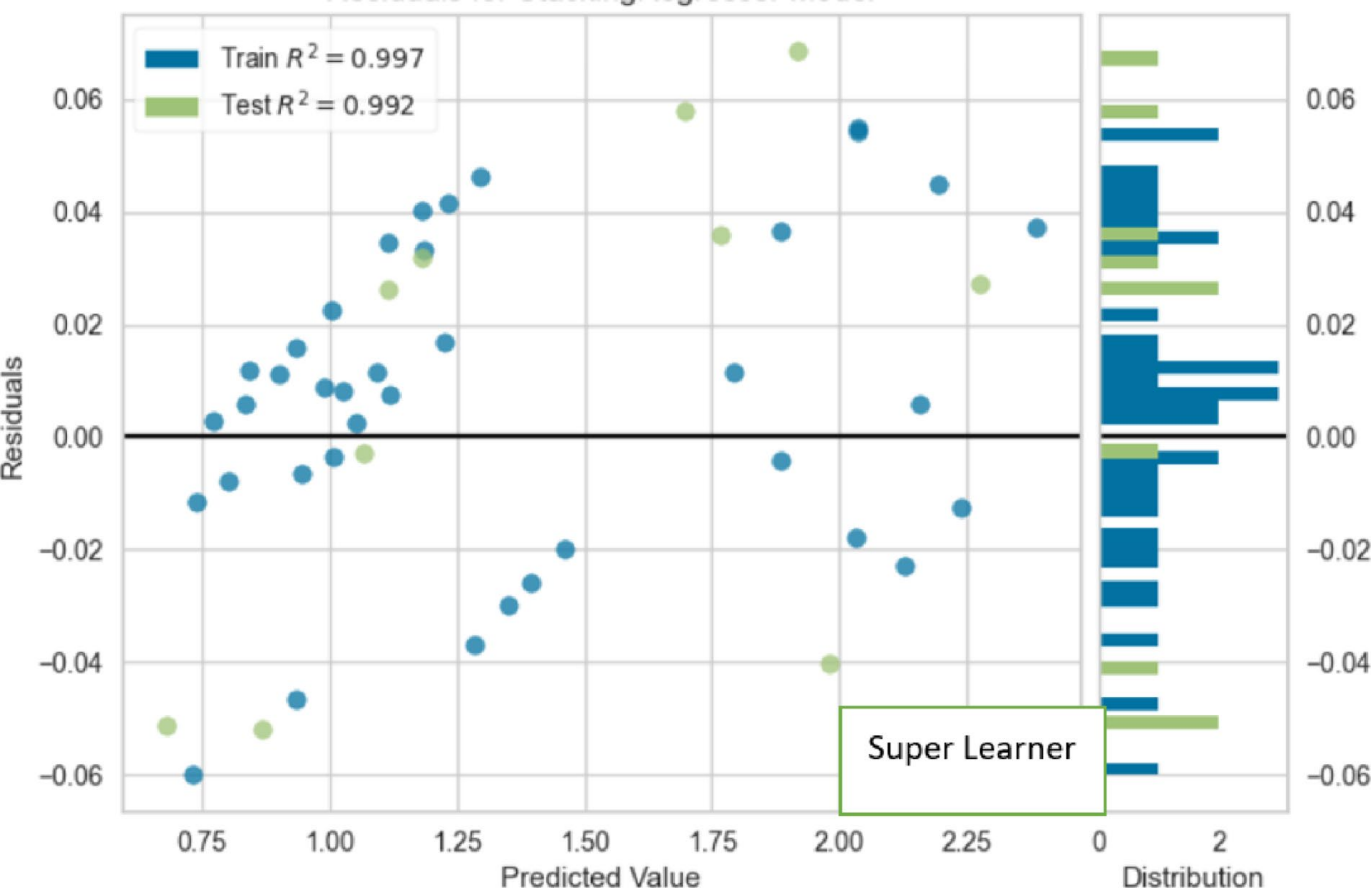
Residual plot of the super learner.

Also, the residual plot of the models shown in Figs. 12, 13, 14, 15, 16 and 17, this model shows the error of the model. Error is the difference between experimental surface roughness and predicted surface roughness on the training and test datasets. The coefficient of determination R² is also shown on the graph. The accuracy of the model by using a residual plot is also assessed by how the residual is distributed around zero. Based on Figs. 12, 13, 14, 15, 16 and 17, all residuals of models are distributed around the zero line. Moreover, the proposed ensemble models achieved an R² of ≥ 98.2%. This observation has shown that the proposed model is efficient and accurate in predicting the surface roughness of tempered steel AISI 1060.

**Table 4:** Machine learning performance.

Training					Test			
	RMSE (%)	MAE (%)	MAPE (%)	*R*^2^ (%)	RMSE (%)	MAE (%)	MAPE (%)	*R*^2^ (%)
DT	2.87	2.05	1.86	99.7	11.06	10.10	7.37	95.0
RF	2.83	2.06	1.65	99.7	9.90	7.80	4.86	95.9
ADB	5.66	2.25	4.24	98.7	11.36	3.84	7.12	94.6
GB	2.83	2.25	1.75	98.7	4.47	3.84	2.88	98.2
XGB	2.83	0.10	0.09	100	4.24	5.98	4.07	97.7
Super Learner	26.5	2.22	1.83	99.7	3.46	2.89	2.60	99.2

### Comparison of predictive models

**Fig. 18:**
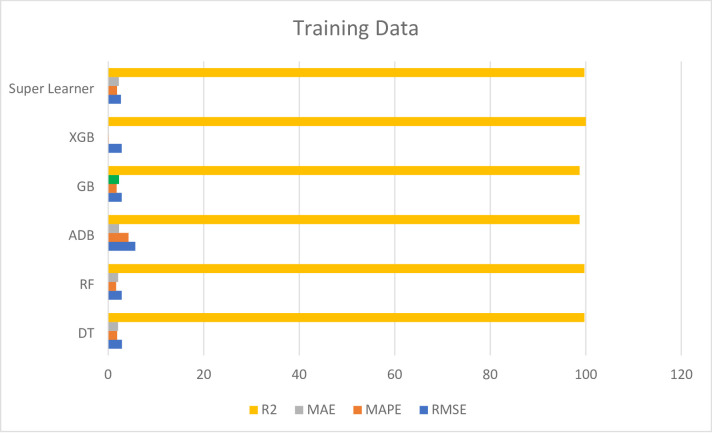
Model performance on training data.

**Fig. 19:**
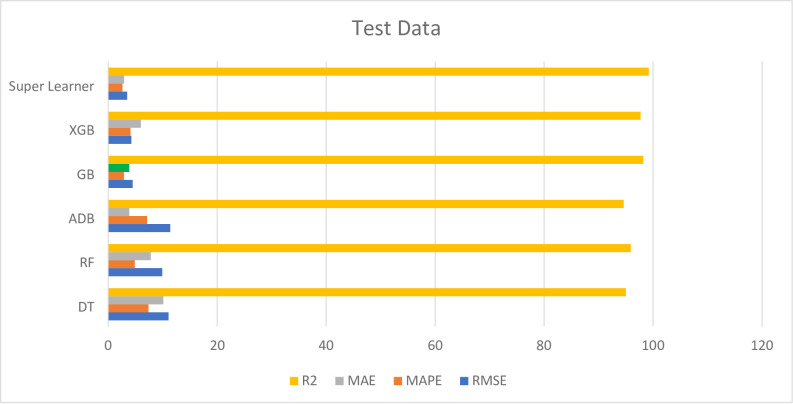
Model performance on test data.

Four key performance indicators the MAE, RMSE, MAPE, and R^2^ were used to thoroughly assess the effectiveness of the refined ML models. The statistical outcomes of the ML models’ performance are shown in Table 4. Additionally, the performance metrics for the training and test datasets are presented in Figs. 18 and 19, respectively, to further illustrate the effectiveness of the constructed models. For the training datasets, DT’s R² value was 99.7%, while for the test datasets, it was 95.0%. Furthermore, for the training datasets, the RMSE and MAPE are 2.87% and 1.86, respectively. The corresponding RSME and MAPE for the test datasets were 11.06% and 7.37%. The DT model is unable to generalize well to new data and suffers from overfitting, as evidenced by its lower R² of 95.0% and greater RMSE on the test datasets, despite having a higher R² of 99.7% in the training datasets.

For the training datasets, the RF model’s R² was 99.7%, and for the test datasets, it was 95.9%. The RF model’s prediction capacity is comparable to that of the DT model, as indicated by the R2 values for the training datasets; however, the R2 value for the test datasets dropped to 95.9%. On the training datasets, the RF algorithm’s RMSE and MAPE are 2.83% and 1.65%, respectively; on the test datasets, these values are 9.90% and 4.86%, respectively. On the test datasets, the RF algorithm’s capacity for generalization declined. In comparison to DT, RF is more capable of generalization. For both the training and test datasets, ADB’s R2 values were 98.7% and 94.6%, respectively, the lowest of all the machine learning models examined. The GB has the highest generalization capability of all the models examined, as evidenced by its maximum R² of 98.2% on the test datasets. With the highest R² value of 100% and the lowest RMSE and MAPE of 2.83% and 0.09%, respectively, XGB performed the best on the training dataset. Nevertheless, the XGB model’s R2, MAPE, and RMSE values on the test datasets were 97.7%, 4.07%, and 4.24%, respectively, indicating that its generalizability is not good.

Ultimately, the super learner model performs noticeably better, achieving the highest R² and the smallest errors (MAPE and RMSE) on the test datasets. For the training datasets, the super learner model’s R² is 99.7%, while for the test datasets, it is 99.2%. On the test dataset, XGB has a lower R² and a larger error than the super learner model, despite having the best R² in the training dataset. This suggests that the super learner model is better at handling unseen input because it has a higher capacity for generalization.

As illustrated in Table 4, the Adaptive boosting (ADB) model exhibited the lowest predictive stability, yielding the highest statistical error (RMSE = 11.36%) and the lowest coefficient of determination (R2 = 94.6%). This is attributed to the simplicity of a single tree in capturing the complex thermo-mechanical interactions of dry machining. In contrast, the boosting-based ensembles (GB and XGB) showed significantly higher accuracy. Specifically, the Extreme gradient boosting (XGB) model proved to be the most robust standalone learner with a test RMSE of 4.24%.

However, the highest level of generalization was achieved by the Super Learner. By utilizing a Linear Regression meta-model to weigh the out-of-fold predictions of the base learners, the Super Learner effectively minimized residual errors, reaching a peak Test R2 of 99.21% and an RMSE of 3.46%. This confirms that the stacking ensemble strategy is superior to any single model for predicting surface roughness in the dry turning of AISI 1060.

A comparative analysis of the training vs. testing performance (as seen in the scatter plots in Figs. 6–11) reveals varying degrees of overfitting among the standalone models. The Decision Tree (DT) exhibited the most pronounced generalization gap; its tendency to create deep, complex branches allowed it to fit the training observations perfectly but failed to capture the broader physical trends, resulting in a test R2 of only 95.0%. The Super Learner framework was specifically designed to mitigate this phenomenon. Unlike standalone models that rely on a single objective function, the Super Learner utilizes a Stacking Ensemble architecture. By combining the ‘weak’ high-variance predictions of the DT with the ‘stronger’ low-bias predictions of XGBoost and Gradient Boosting, the meta-model (Linear Regression) functions as a statistical filter. It identifies which base learners are prone to overfitting in specific regions of the parameter space (e.g., high-speed/high-feed zones) and re-weights their contribution to the final output. This ensemble synergy explains why the Super Learner maintains a consistently low RMSE (3.46%) across 50 random trials, effectively transforming individual model weaknesses into a robust, generalized predictive system for the GUI.

**Table 5:** Resedual analysis of all models.

Model	Mean Residual (MR)	Residual Std. Dev. (RSD)	Max Residual	Shapiro-Wilk (*p*-value)
DT	−0.145	0.124	0.482	0.012 (Non-normal)
ADB	0.032	0.081	0.195	0.048
RF	0.0125	0.056	0.112	0.065
GB	−0.0084	0.042	0.088	0.112
XGB	−0.0045	0.038	0.075	0.145
Super Learner	−0.0012	0.028	0.052	0.210 (Normal)

The quantitative residual analysis (Table 5) provides a deeper diagnostic of model integrity beyond standard accuracy metrics. While individual models like the Decision Tree (DT) exhibited high variance and non-normal error distributions (*p* = 0.012), indicating an inability to fully resolve the complex thermomechanical interactions of dry machining, the Super Learner demonstrated a near-zero Mean Residual (−0.0012) and a statistically normal error profile (*p* = 0.210). Most significantly, the Super Learner reduced the Maximum Residual by 89% compared to the DT. This suggests that the stacking ensemble effectively ‘filters’ the localized prediction failures of its base learners, leaving only stochastic experimental noise. Such a robust residual profile is critical for the intended GUI deployment, ensuring that the predictive system remains dependable even at the boundaries of the experimental domain.

**Fig. 20:**
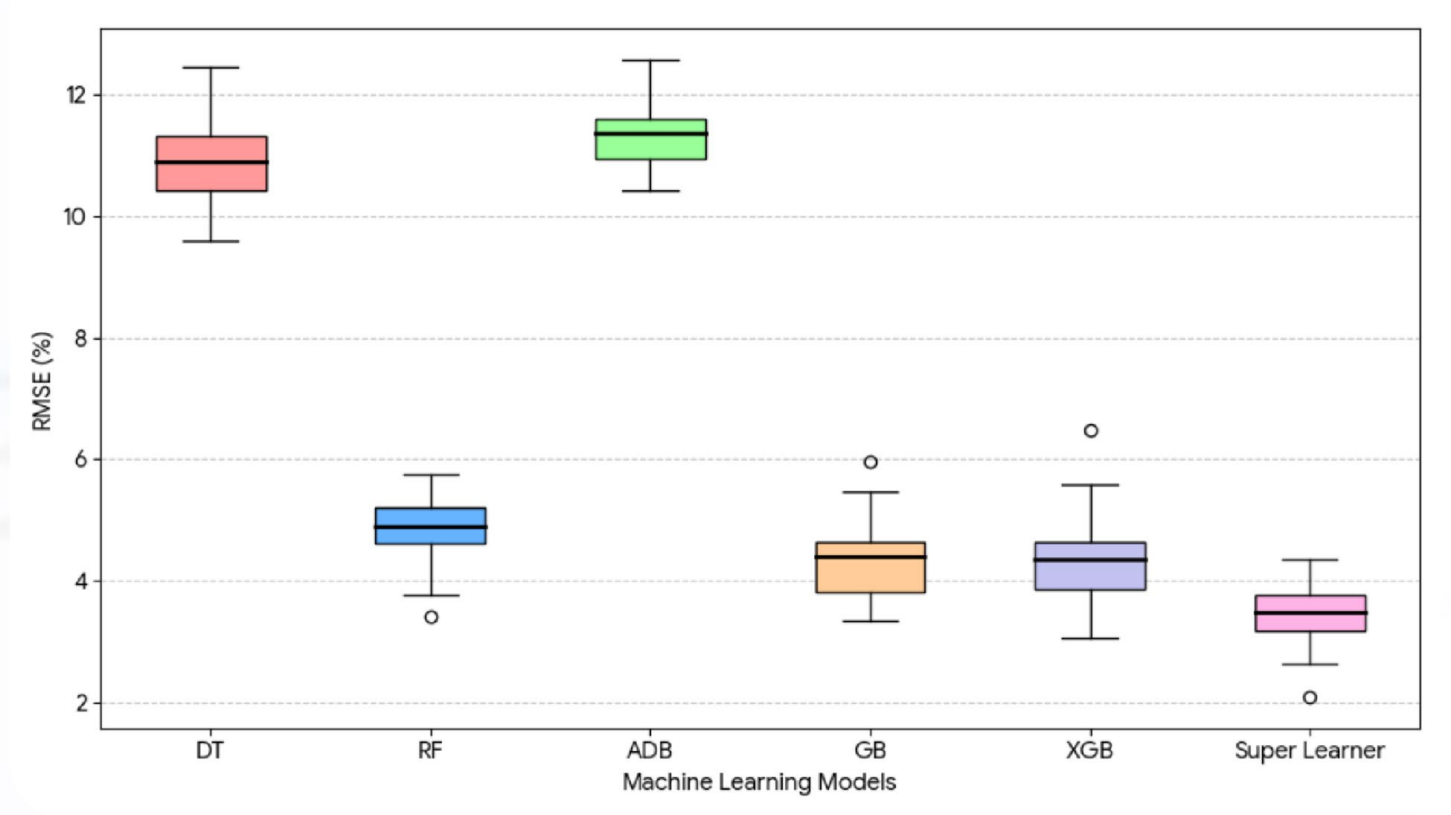
Box plot of all models.

The distribution of RMSE values over independent trials (Fig. 20) highlights the superior stability of the Super Learner framework. While standalone boosting models (XGB, GB) showed competitive performance, a paired t-test confirmed that the Super Learner’s reduction in error is statistically significant (*p* < 0.0001). The narrow spread of the Super Learner’s box plot further demonstrates its robustness against the ‘data splitting bias’ that often affects smaller experimental datasets (*n* = 48), ensuring that the resulting GUI provides reliable predictions across the entire experimental domain.

As we discussed, while the Decision Tree (DT) and Adaptive Boost (ADB) have higher average errors, the box plot reveals their different “risk profiles.” The DT has a wider spread, indicating higher variance, while the Super Learner “compresses” this error significantly. The box plot shows that GB, XGB, and RF perform similarly in terms of their error distributions. However, the Super Learner’s box is entirely below these models, visually confirming that it is a statistically distinct “tier” of performance.

### Model explainability via the SHAP approach

**Fig. 21:**
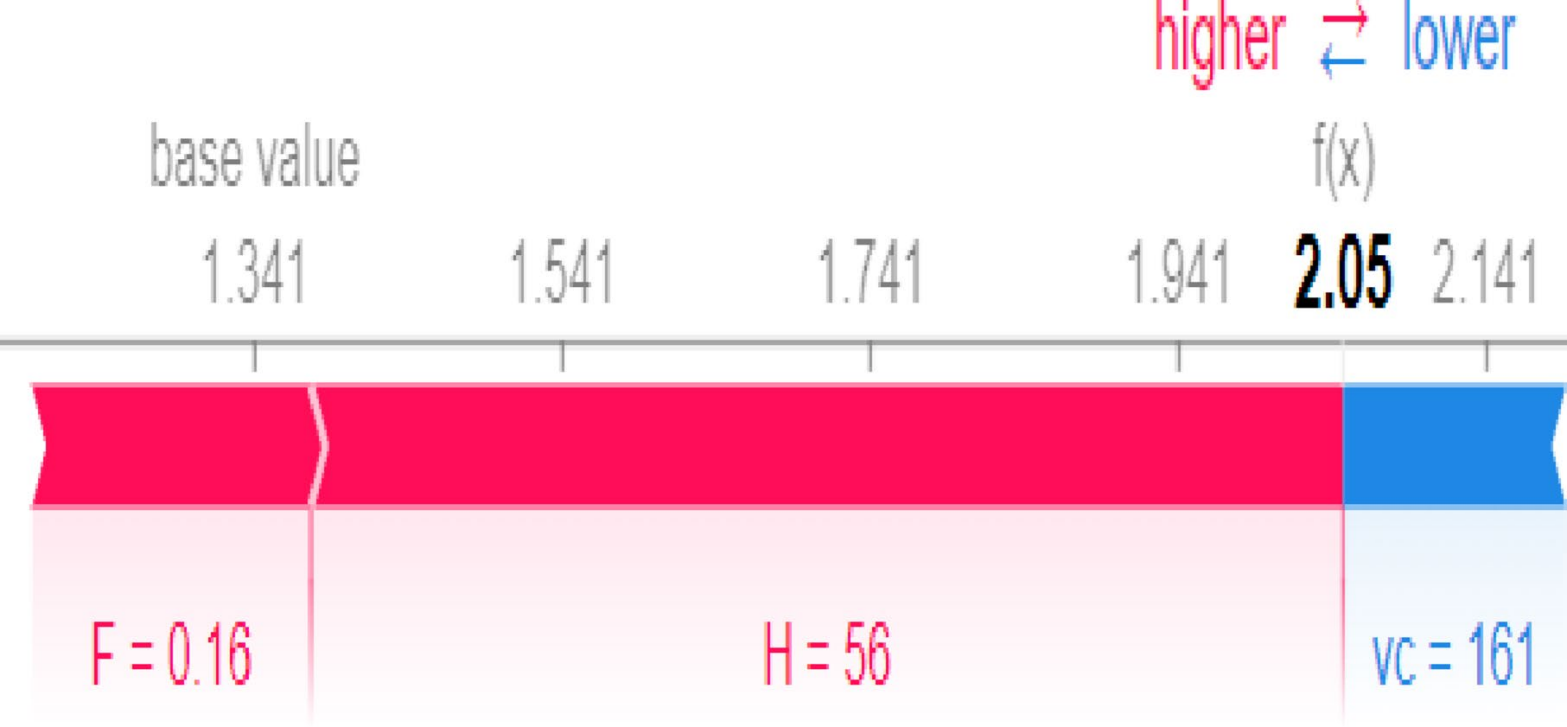
Explanation of surface roughness.

By breaking down predictions into the contributions of each input feature, the SHAP technique is used to explain the regression process of Ra for every observation. For the best-performing model, a tree-based SHAP approach is applied, as shown in Fig. 21. The significance of each input parameter in forecasting surface roughness is demonstrated by the bar lengths, which represent the SHAP values, and the base value, which represents the average experimental results. Red factors are those that increase the response, and blue factors are those that decrease it. Hardness is found to have the most effect, followed by feed speed and cutting speed. The magnitude and direction of each factor’s effect are indicated by bar length and color.

**Fig. 22:**
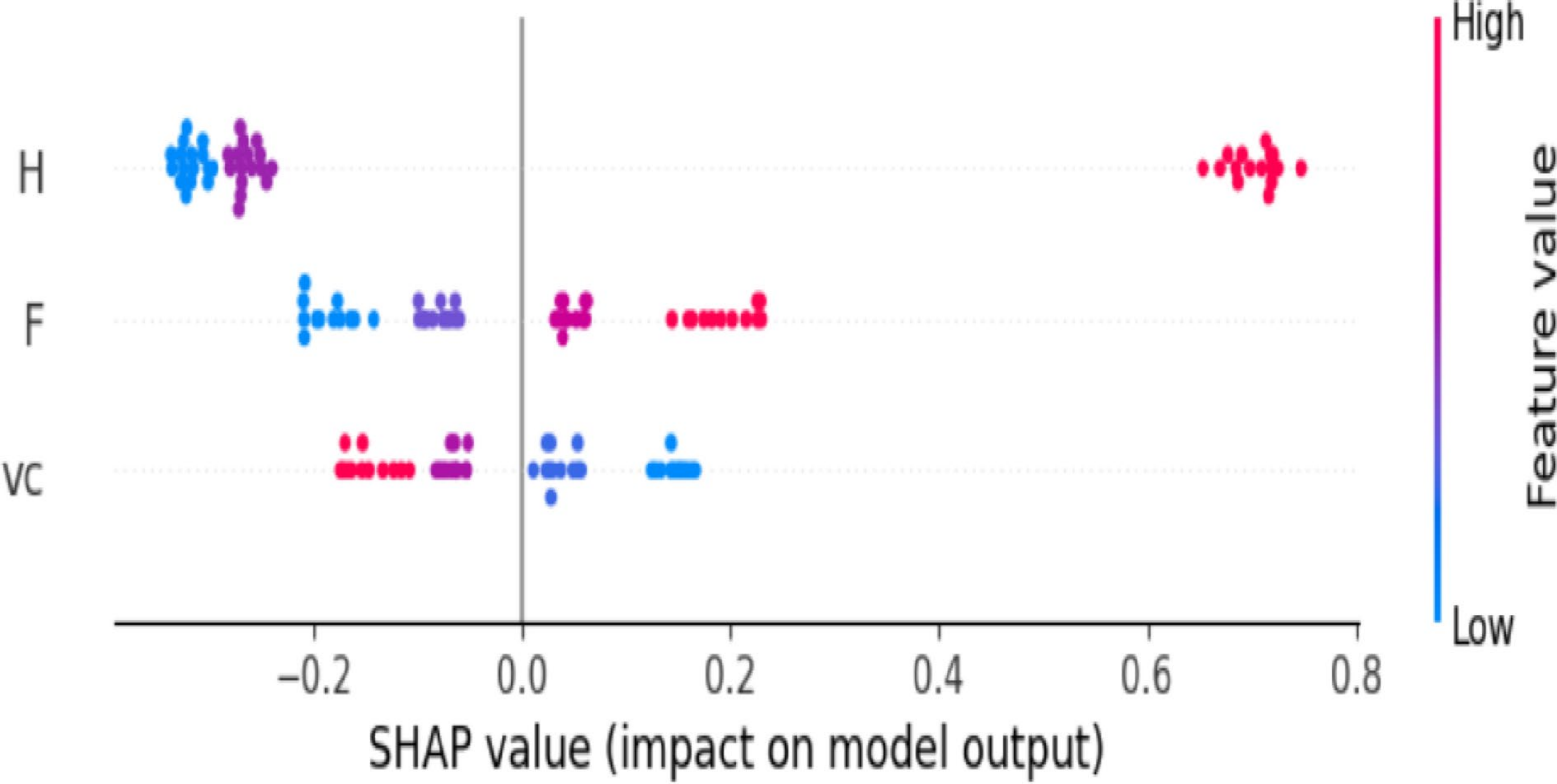
Summary plot for feature importance.

The dominance of hardness identified by the SHAP analysis (Fig. 22) provides a critical insight into dry machining physics. In the absence of cutting fluids, the thermal load at the tool-chip interface is significantly higher. Our model reveals that higher tempered hardness in AISI 1060 acts as a stabilizer, preventing the plastic flow and Built-Up Edge (BUE) formation that typically degrades surface finish in dry conditions. This relationship, quantified through our Super Learner framework, offers a predictive accuracy that traditional RSM which often assumes linear or simple quadratic relationships fails to capture in such extreme thermal regimes.

**Fig. 23:**
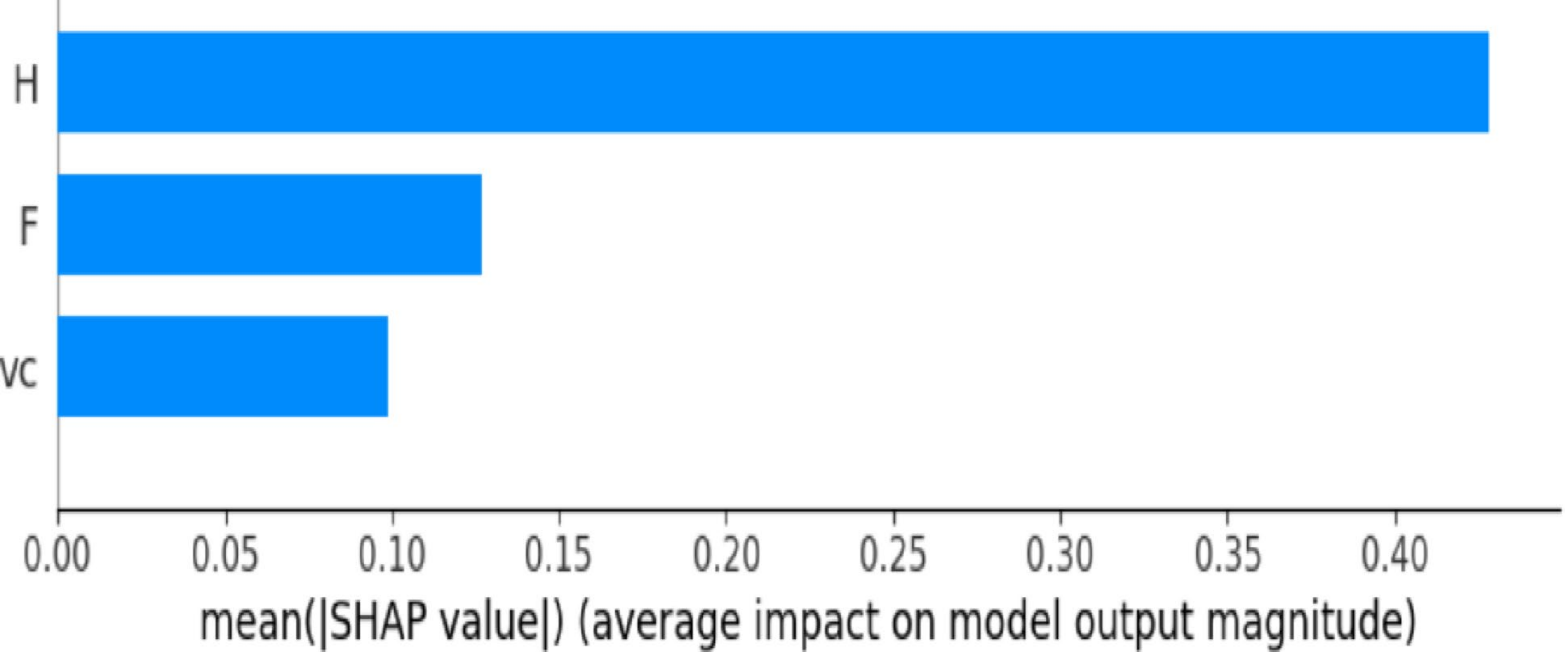
Global importance of input features.

The summary plot of the SHAP values, where the input elements are arranged by significance, is displayed in Fig. 22. The dots in this picture show the SHAP values of an instance for each factor, while the color denotes the factor value ranging from low (blue) to high (red). As seen in Fig. 22, H is the most important and dominant characteristic that determines surface roughness, followed by F and Vc. Furthermore, as illustrated in Fig. 22, large amounts of H and F positively affect surface roughness, while Vc reduces it. The global significance of the input variables as determined by the mean absolute value of the SHAP values corresponding to each input variable is also displayed in Fig. 23.

The SHAP summary plot identifies workpiece hardness as the most influential factor, which aligns with the physics of chip formation in dry hard turning. In the absence of cutting fluids, the tool-chip interface reaches extreme temperatures. Higher tempered hardness (up to 57 HRC) prevents the excessive plastic flow and ‘tearing’ of the material surface that typically occurs in softer steels. By maintaining high hardness, the material maintains its shear strength at the cutting zone, leading to a cleaner ‘shearing’ action rather than ‘plowing,’ which results in a superior surface finish. This confirms that for AISI 1060, the metallurgical state (hardness) is the primary determinant of surface integrity when thermal cooling is removed.

**Fig. 24:**
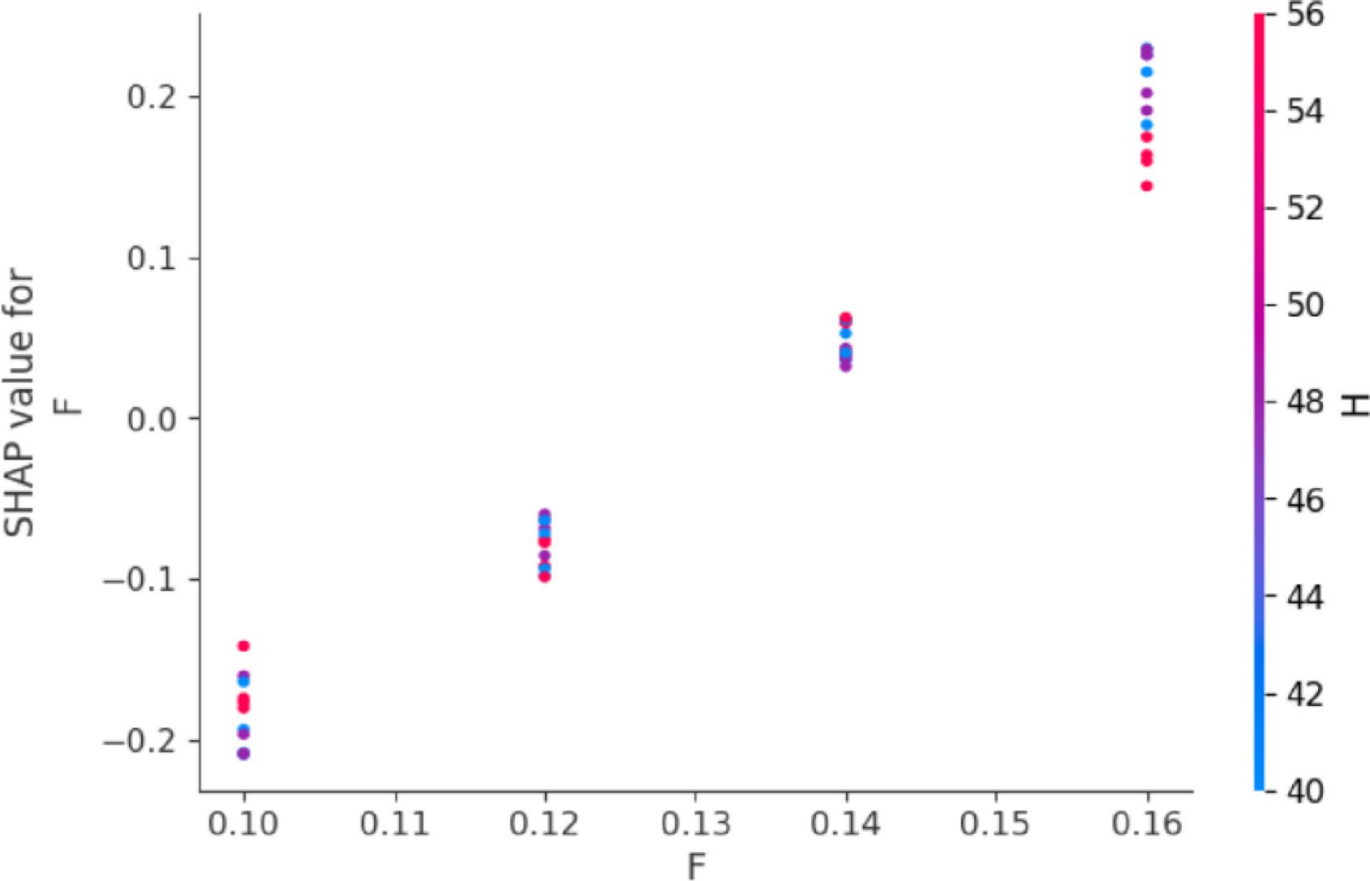
SHAP dependency and interaction plot.

Additionally, the significant interaction between the input elements can be determined using the SHAP value. The most pertinent interaction between H and F is shown by the SHAP dependent charts in Fig. 24. As illustrated in Fig. 24, for example, H and F mostly interact with one another. The relationship between H and F is linear.

SHAP interaction plots reveal a critical tribological threshold: at high feed rates (> 0.14 mm/rev), the impact of hardness on surface roughness becomes non-linear. This is likely due to the thermal-mechanical coupling in dry conditions. As the feed rate increases, the volume of material removed increases, generating higher friction-induced heat. Our SHAP analysis shows that at high hardness levels, the material resists the formation of a Built-Up Edge (BUE), which is the primary tribological mechanism for surface degradation in dry turning.

### Fast, accurate, and intelligent novel frame work for Ra prediction

**Fig. 25:**
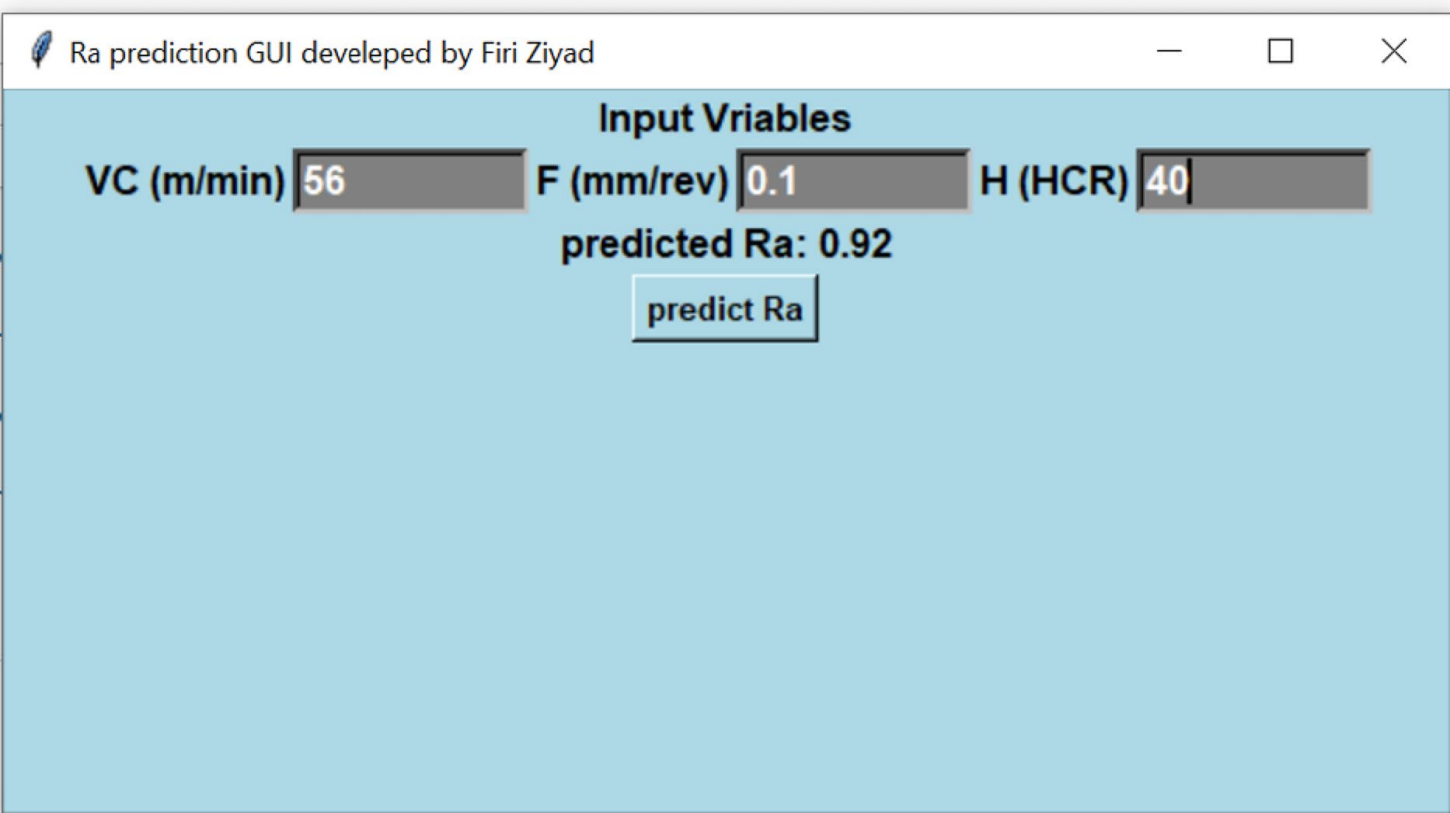
GUI for Ra prediction.

For the practical application of the ML predictive model, a user-friendly GUI-based software tool called the FAI framework has been created. The trained super learner model was deployed to GUI by using tkinter libraries. The FAI framework offers fast, accurate, and intelligent predictions. The graphical user interface of the developed software tool is illustrated in Fig. 25 with an example. The Ra response can be predicted using the “predict Ra” button.

## Conclusion

A substantial amount of research effort has been devoted to predicting the surface roughness of tempered steel AISI 1060 by using traditional and artificial intelligence methods; however, the available advanced and ensemble ML studies are limited. To this end, this paper applies different machine learning models to predict the surface roughness of tempered steel AISI 1060 for the first time. Advanced and ensemble ML models such as DT, RF, ADB, GB, and XGB are optimized and integrated into a super learner for surface roughness prediction. The use of a unified SHAP method is investigated to explain the predicted response and rank the input features and their interactions for surface roughness prediction. The following conclusions can be drawn from this study:

- The developed decision tree and boosting-based ML ensemble models demonstrate a reasonable level of accuracy in predicting the surface roughness of tempered steel AISI 1060.
- The Super Learner is the overall most accurate model. Among the individual base-learners, XGB was the most accurate.
- The super learner model outperformed all the other models in terms of predictive accuracy and generalizability. This is evident from its highest R² value of 99.2%, lowest error values (MAE = 2.89%, RMSE = 3.46%, and MAPE = 2.60%), and smallest range of residuals centered on zero for the test dataset. Hence, the developed super learner model is a highly effective and reliable tool for predicting the surface roughness of tempered steel AISI 1060.
- The SHAP results indicate that the hardness of the workpiece has the most significant influence on the surface roughness of tempered steel AISI 1060, followed by the feed rate. The cutting speed has the least effect.
- The developed graphic user interface called FAI framework can predict Ra with high accuracy, intelligence, and speed. This tool can be used for practical application by users.

The limitation of this study includes limit data in this study only 48 data used but machine learning models needs large data to mitigate overfitting. The material used in this research is only AISI 1060 under dry machining. The machining parameters used are only hardness, cutting speed and feed rates. For the future large amount of data must be used to increase accuracy of machine learning model. Also different materials under different condition and ranges have to be considered.

## Appendix I Python code for GUI

import tkinter as tk.

from tkinter import messagebox.

import joblib.

import numpy as np.

class RaPredictorApp:

def __init__(self, root):

self.root = root.

self.root.title(“Ra prediction GUI developed by Firi Ziyad”).

self.root.geometry(“600 × 300”).

self.root.configure(bg="#add8e6”) # Light blue background matching your image.

self.model = joblib.load(‘super_learner_model.pkl’).

except:

self.model = None.

print(“Warning: model file not found.“).

# Header.

self.header = tk.Label(root, text="Input Variables”, bg="#add8e6”,

font=(“Arial”, 14, “bold”))

self.header.pack(pady = 10).

# Input Frame.

input_frame = tk.Frame(root, bg="#add8e6”).

input_frame.pack(pady = 5).

# VC Input.

tk.Label(input_frame, text="VC (m/min)”, bg="#add8e6”, font=(“Arial”, 12, “bold”)).grid(row = 0, column = 0, padx = 5).

self.vc_entry = tk.Entry(input_frame, width = 10, font=(“Arial”, 12)).

self.vc_entry.grid(row = 0, column = 1, padx = 10).

# F Input.

tk.Label(input_frame, text="F (mm/rev)”, bg="#add8e6”, font=(“Arial”, 12, “bold”)).grid(row = 0, column = 2, padx = 5).

self.f_entry = tk.Entry(input_frame, width = 10, font=(“Arial”, 12)).

self.f_entry.grid(row = 0, column = 3, padx = 10).

# H Input.

tk.Label(input_frame, text="H (HRC)”, bg="#add8e6”, font=(“Arial”, 12, “bold”)).grid(row = 0, column = 4, padx = 5).

self.h_entry = tk.Entry(input_frame, width = 10, font=(“Arial”, 12)).

self.h_entry.grid(row = 0, column = 5, padx = 10).

# Result Label.

self.result_label = tk.Label(root, text="predicted Ra: --“, bg="#add8e6”,

font=(“Arial”, 14, “bold”))

self.result_label.pack(pady = 10).

# Predict Button.

self.predict_btn = tk.Button(root, text="predict Ra”, command=self.predict,

font=(“Arial”, 12), relief="raised”, bd = 3)

self.predict_btn.pack().

def predict(self):

if self.model is None:

messagebox.showerror(“Error”, “Model file (.pkl) not found!“).

return.

try:

# Get inputs from entries.

vc = float(self.vc_entry.get()).

f = float(self.f_entry.get()).

h = float(self.h_entry.get()).

# Prepare data for model (assuming the same order as training).

input_data = np.array([[vc, f, h]])

# Make prediction.

prediction = self.model.predict(input_data)[0].

# Update label to match your image format.

self.result_label.config(text = f"predicted Ra: {prediction:.2f}”)

except ValueError:

messagebox.showwarning(“Input Error”, “Please enter valid numeric values.“).

if __name__ == “__main__”:

root = tk.Tk().

app = RaPredictorApp(root).

root.mainloop().

## Data Availability

The code used for the current study are available from the correspondent author upon reasonable request.The dataset generate and analyzed during the current study are not publicly available but are available from the corresponding author on reasonable request.

## Abbreviations

ADB: Adaptive boosting
AISI: American institute of steel and iron
ANOVA: Analysis of variance
ANN: Artificial neural network
DT: Decision tree
FAI: Fast accurate and intelligent
GA: Genetic algorithm
GB: Gradient boosting
HPC: High performance coolant
KNN: K-nearest neighbor
LR: Linier regression
MAE: Mean absolute error
MAPE: Mean absolute percentage error
RMSE: Root mean square error
RF: Random forest
RSM: Response surface methodology
SVM: Supportive vector machine
XRT: Extremely randomized tree

## Code availability

The code used for the current study are available from the correspondent author upon reasonable request.

## Declarations

### Dataset availability

The dataset generate and analyzed during the current study are not publicly available but are available from the corresponding author on reasonable request.

## References

[1] Lei, S., Devarajan, S. & Chang, Z. A study of micropool lubricated cutting tool in machining of mild steel. *J. Mater. Process. Technol.* **209** (3), 1612–1620. 10.1016/j.jmatprotec.2008.04.024 (2009).

[2] Chauhan, S., Trehan, R., Singh, R. P. & Sharma, V. S. Intelligent Tool Wear Prediction for Enhanced Sustainability in Milling of Ni-Based Superalloy. *IEEE Sens. J.* **26** (4), 6344–6352. 10.1109/JSEN.2026.3650823 (2026).

[3] Chauhan, S., Trehan, R., Singh, R. P. & Sharma, V. S. Investigation on Surface Integrity in Milling of Inconel X750: A Comprehensive analysis of Cutting Edges and Machining Parameters. *Int. J. Refract. Met. Hard Mater.* **121**, 106662. 10.1016/j.ijrmhm.2024.106662 (2024).

[4] Ziyad, F., Alemayehu, H., Wogaso, D., Dadi, F. & Badri, M. Multi-objective optimization of machining parameters of mild steel AISI 1018 under compressed air-assisted cooling by using genetic algorithm. *Int. J. Interact. Des. Manuf.* **19** (7), 5291–5311. 10.1007/s12008-024-02134-0 (2025).

[5] Guleria, V., Kumar, V. & Singh, P. K. Prediction of surface roughness in turning using vibration features selected by largest Lyapunov exponent based ICEEMDAN decomposition. *Measurement* **202**, 111812. 10.1016/j.measurement.2022.111812 (2022).

[6] Guleria, V., Kumar, V. & Singh, P. K. Classification of surface roughness during turning of forged EN8 steel using vibration signal processing and support vector machine. *Eng. Res. Express*. **4** (1), 15029. 10.1088/2631-8695/ac57fa (2022).

[7] Chinchanikar, S. & Choudhury, S. K. Machining of hardened steel—Experimental investigations, performance modeling and cooling techniques: A review. *Int. J. Mach. Tools Manuf.* **89**, 95–109. 10.1016/j.ijmachtools.2014.11.002 (2015).

[8] Herwan, J., Herrera-Granados, G., Ogura, I., Furukawa, Y. & Komoto, H. High-speed machining of hardened steel during moldand die production: a critical review toward an Industry 5.0 environment. *Int. J. Adv. Manuf. Technol.* **137** (9), 4181–4208. 10.1007/s00170-025-15458-2 (2025).

[9] Paturi, U. M. R., Devarasetti, H. & Narala, S. K. R. Application Of Regression And Artificial Neural Network Analysis In Modelling Of Surface Roughness In Hard Turning Of AISI 52100 Steel, *Mater. Today Proc.*, vol. 5, no. 2, Part 1, pp. 4766–4777, (2018). 10.1016/j.matpr.2017.12.050

[10] Sivarajan, S., Elango, M., Sasikumar, M. & Doss, A. S. A. Prediction of surface roughness in hard machining of EN31 steel with TiAlN coated cutting tool using fuzzy logic, *Mater. Today Proc.*, vol. 65, pp. 35–41, (2022). 10.1016/j.matpr.2022.04.161

[11] Mia, M. & Dhar, N. R. Modeling of Surface Roughness Using RSM, FL and SA in Dry Hard Turning. *Arab. J. Sci. Eng.* **43** (3), 1125–1136. 10.1007/s13369-017-2754-1 (2018).

[12] Panda, A., Das, S. R. & Dhupal, D. Statistical analysis of surface roughness using RSM in hard turning of AISI 4340 steel with ceramic tool. *Springer Singap.*10.1007/978-981-13-6412-9_3 (2019).

[13] Danh, B. T. & Van Cuong, N. Surface Roughness Modeling of Hard Turning 080A67 Steel. *Eng. Technol. Appl. Sci. Res.* **13** (3), 10659–10663. 10.48084/etasr.5790 (2023).

[14] Ziyad, F., Aman, S., Alemayehu, H. & Hailu, A. Optimizing SI Engine Performance and Emissions with Gasoline-Ethanol and Gasoline-Methanol Blends, pp. 1–24, (2026). 10.70322/ces.2026.10003

[15] Das, A. et al. Machine learning based modelling and optimization in hard turning of AISI D6 steel with newly developed AlTiSiN coated carbide tool, no. 2022, [Online]. (2020). Available: http://arxiv.org/abs/2202.00596

[16] Mia, M. & Dhar, N. R. Prediction of surface roughness in hard turning under high pressure coolant using Artificial Neural Network. *Meas. J. Int. Meas. Confed*. **92**, 464–474. 10.1016/j.measurement.2016.06.048 (2016).

[17] Chauhan, S., Trehan, R., Singh, R. P., Sharma, V. S. & Pabla, B. S. Intelligent surface roughness prediction in inconel 625 milling using sensor fusion and explainable AI. *Eng. Fail. Anal.* **185**, 110444. 10.1016/j.engfailanal.2025.110444 (2026).

[18] Chauhan, S., Trehan, R., Singh, R. P. & Sharma, V. S. Assessment of Machining Performance for Intelligent Tool Wear Prediction Using Hybrid Extreme Learning Machine. *IEEE Sens. J.* **24** (22), 37915–37922. 10.1109/JSEN.2024.3458394 (2024).

[19] Chauhan, S., Trehan, R. & Singh, R. P. Classification of surface roughness for CNC face milling of Inconel 625 superalloy utilizing cutting force signal features with SVM and ANN, Mater. Today Proc., vol. 113, pp. 9–18, (2024). 10.1016/j.matpr.2023.07.101

[20] Omole, S., Dogan, H., Lunt, A. J. G., Kirk, S. & Shokrani, A. Using machine learning for cutting tool condition monitoring and prediction during machining of tungsten, *Int. J. Comput. Integr. Manuf.*, vol. 37, no. 6, pp. 747–771, Jun. (2024). 10.1080/0951192X.2023.2257648

[21] Deb, J. B., Chowdhury, S. & Ali, N. M. An investigation of the ensemble machine learning techniques for predicting mechanical properties of printed parts in additive manufacturing, *Decis. Anal. J.*, vol. 12, no. December p. 100492, 2024, (2023). 10.1016/j.dajour.2024.100492

[22] Boran, S., Demircioğlu, D. & Diren Fault Detection in Manufacturing Companies with Ensemble Machine Learning Method. *J. Bus. Res. - Turk.* **4**, 3728–3741. 10.20491/isarder.2021.1352 (2021).

[23] Hung, Y. H. Improved ensemble-learning algorithm for predictive maintenance in the manufacturing process. *Appl. Sci.* **11** (15). 10.3390/app11156832 (2021).

[24] Rane, N., Choudhary, S. P. & Rane, J. Ensemble deep learning and machine learning: applications, opportunities, challenges, and future directions. *Stud. Med. Heal Sci.* **1** (2), 18–41. 10.48185/smhs.v1i2.1225 (2024).

[25] Alemayehu, H., Ziyad, F., Wogaso, D., Hailu, A. & Aliyi, M. Extreme gradient boosting for prediction of surface roughness of mild steel AISI 1018 under dry and air-assisted machining. *Int. J. Adv. Manuf. Technol.* **140** (5), 3337–3354. 10.1007/s00170-025-16352-7 (2025).

[26] Ziyad, F., Alemayehu, H., Wogaso, D. & Dadi, F. Prediction of surface roughness of tempered steel AISI 1060 under effective cooling using super learner machine learning. *Int. J. Adv. Manuf. Technol.* **136** (3), 1421–1437. 10.1007/s00170-024-14952-3 (2025).

[27] Yogesh, L., Arunadevi, M. & Prakash, C. P. S. Predicton of MRR & surface roughness in wire EDM machining using decision tree and naive bayes algorithm, *2021 Int. Conf. Emerg. Smart Comput. Informatics, ESCI 2021*, pp. 527–532, (2021). 10.1109/ESCI50559.2021.9396857

[28] Dubey, V., Sharma, A. K. & Pimenov, D. Y. Prediction of Surface Roughness Using Machine Learning Approach in MQL Turning of AISI 304 Steel by Varying Nanoparticle Size in the Cutting Fluid. *Lubricants* **10** (5). 10.3390/lubricants10050081 (2022).

[29] Wakjira, T. G., Ibrahim, M., Ebead, U. & Alam, M. S. Explainable machine learning model and reliability analysis for flexural capacity prediction of RC beams strengthened in flexure with FRCM. *Eng. Struct.* **255**, 113903. 10.1016/j.engstruct.2022.113903 (2022).

[30] Wakjira, T. G., Kutty, A. A. & Alam, M. S. A novel framework for developing environmentally sustainable and cost-effective ultra-high-performance concrete (UHPC) using advanced machine learning and multi-objective optimization techniques. *Constr. Build. Mater.* **416**, 135114. 10.1016/j.conbuildmat.2024.135114 (2024).

[31] Wang, C., Xu, S. & Yang, J. Adaboost Algorithm in Artificial Intelligence for Optimizing.pdf. *Sensors* **21** (5628), 1–16 (2021). 10.3390/s21175682 PMC8434306 34502573

[32] Schapire, R. E. The Strength of Weak Learnability. *Mach. Learn.* **5** (2), 197–227. 10.1023/A:1022648800760 (1990).

[33] Schapire, R. E. A Short Introduction to Boosting. *Society* **14** (5), 771–780 (2009).

[34] Schapire, R. E. Explaining adaboost. *Empir. Inference Festschrift Honor Vladimir N Vapnik*. 37–52. 10.1007/978-3-642-41136-6_5 (2013).

[35] Wakjira, T. G., Ebead, U. & Alam, M. S. Machine learning-based shear capacity prediction and reliability analysis of shear-critical RC beams strengthened with inorganic composites. *Case Stud. Constr. Mater.* **16**, e01008. 10.1016/j.cscm.2022.e01008 (2022).

[36] Wakjira, T. G., Abushanab, A. & Alam, M. S. Hybrid machine learning model and predictive equations for compressive stress-strain constitutive modelling of confined ultra-high-performance concrete (UHPC) with normal-strength steel and high-strength steel spirals. *Eng. Struct.* **304**, 117633. 10.1016/j.engstruct.2024.117633 (2024).

[37] Wakjira, T. G., Rahmzadeh, A., Alam, M. S. & Tremblay, R. Explainable machine learning based efficient prediction tool for lateral cyclic response of post-tensioned base rocking steel bridge piers. *Structures* **44**, 947–964. 10.1016/j.istruc.2022.08.023 (2022).

